# Gut microbiome for predicting immune checkpoint blockade-associated adverse events

**DOI:** 10.1186/s13073-024-01285-9

**Published:** 2024-01-19

**Authors:** Muni Hu, Xiaolin Lin, Tiantian Sun, Xiaoyan Shao, Xiaowen Huang, Weiwei Du, Mengzhe Guo, Xiaoqiang Zhu, Yilu Zhou, Tianying Tong, Fangfang Guo, Ting Han, Xiuqi Wu, Yi Shi, Xiuying Xiao, Youwei Zhang, Jie Hong, Haoyan Chen

**Affiliations:** 1 State Key Laboratory of Systems Medicine for Cancer, NHC Key Laboratory of Digestive Diseases, Division of Gastroenterology and Hepatology, School of Medicine, Renji Hospital, Shanghai Jiao Tong University, Shanghai Institute of Digestive Disease, Shanghai Cancer Institute, Shanghai, 200001 China; 2 Department of Oncology, Renji Hospital, Shanghai Jiao Tong University School of Medicine, Shanghai, 200127 China; 3 Department of Medical Oncology, Xuzhou Central Hospital, Clinical School of Xuzhou Medical University, Xuzhou, 221009 China; 4 Jiangsu Key Laboratory of New Drug Research and Clinical Pharmacy, Xuzhou Medical University, Xuzhou, China; 5 Department of Gastroenterology, the First Affiliated Hospital, Zhejiang University School of Medicine, 79 Qingchun Road, Hangzhou, 310003 China; 6 Bio-X Institutes, Key Laboratory for the Genetics of Developmental and Neuropsychiatric Disorders, Shanghai Jiao Tong University, 1954 Huashan Road, Shanghai, 200030 China

**Keywords:** Immune-related adverse events, Gut microbiome, Immune checkpoint inhibitors, Programmed death 1 (PD-1)/programmed death ligand 1 (PD-L1)

## Abstract

**Background:**

The impact of the gut microbiome on the initiation and intensity of immune-related adverse events (irAEs) prompted by immune checkpoint inhibitors (ICIs) is widely acknowledged. Nevertheless, there is inconsistency in the gut microbial associations with irAEs reported across various studies.

**Methods:**

We performed a comprehensive analysis leveraging a dataset that included published microbiome data (*n* = 317) and in-house generated data from 16S rRNA and shotgun metagenome samples of irAEs (*n* = 115). We utilized a machine learning-based approach, specifically the Random Forest (RF) algorithm, to construct a microbiome-based classifier capable of distinguishing between non-irAEs and irAEs. Additionally, we conducted a comprehensive analysis, integrating transcriptome and metagenome profiling, to explore potential underlying mechanisms.

**Results:**

We identified specific microbial species capable of distinguishing between patients experiencing irAEs and non-irAEs. The RF classifier, developed using 14 microbial features, demonstrated robust discriminatory power between non-irAEs and irAEs (AUC = 0.88). Moreover, the predictive score from our classifier exhibited significant discriminative capability for identifying non-irAEs in two independent cohorts. Our functional analysis revealed that the altered microbiome in non-irAEs was characterized by an increased menaquinone biosynthesis, accompanied by elevated expression of rate-limiting enzymes *menH* and *menC*. Targeted metabolomics analysis further highlighted a notably higher abundance of menaquinone in the serum of patients who did not develop irAEs compared to the irAEs group.

**Conclusions:**

Our study underscores the potential of microbial biomarkers for predicting the onset of irAEs and highlights menaquinone, a metabolite derived from the microbiome community, as a possible selective therapeutic agent for modulating the occurrence of irAEs.

**Supplementary Information:**

The online version contains supplementary material available at 10.1186/s13073-024-01285-9.

## Background

Immune checkpoint inhibitors (ICIs), contributing to durable remissions in a subset of cancer patients, have reshaped multiple cancer therapy paradigms over the past several decades [1, 2]. The traditional ICI drugs, including anti-cytotoxic T lymphocyte-associated antigen 4 (CTLA-4) and other checkpoint inhibitors targeting the programmed death 1(PD-1)/programmed death ligand 1 (PD-L1) pathway, were approved for the treatment of multiple cancers [3–5]. Although ICIs were confirmed to boost the tremendous clinical benefit of defense against cancer, they might bring adverse effects, collectively termed as immune-related adverse events (irAEs), that represented autoimmune effects and damage on the normal tissues suffering misdirected overactivation of the immune systems [6]. Common irAEs encroached on organs involving the skin (e.g., rash), the gastrointestinal tract (e.g., emesis or colitis), lungs (e.g., pneumonitis), heart (e.g., myocarditis), liver (e.g., hepatitis), and endocrine organs (e.g., autoimmune diabetes), etc. [7]. These toxicities were important causes of treatment discontinuation and can debilitate long-term clinical responses and life quality [8]. Previous evidence demonstrated that patients who received anti-CTLA-4 therapy suffered from a higher incidence of irAEs than ICIs targeting PD-1/PD-L1 [9]. Moreover, histologically pathologic features of anti-PD-1/PD-L1 therapy-related colitis were distinct from anti-CTLA-4 therapy [10]. These supported the idea that effective biomarkers for irAEs should be specific to the ICI agent used.

Strikingly, patients who experienced irAEs usually had a relatively improved progression-free survival (PFS) and overall survival (OS) compared with those who suffered none of irAEs [11, 12]. Therefore, the effective prevention and treatment of irAEs is a clinical paradox to maximize the utility of ICIs. Oncologists must weigh the risk of irAE onsets against the benefit of ICIs before prescribing ICI agents. Thus, intensive efforts were encouraged to identify potential biomarkers for the occurrence and severity of irAEs, thereby guiding the rational medications and surveillance strategies for high-risk patients allowing for earlier intervention [13].

Emergent studies have struggled to investigate the potential mechanisms and related strategies to recognize the patients who might be susceptible to suffering from irAEs. However, lack of scientific rigor and reproducibility, in concert with complex laboratory diagnosis and limitative study scale, prevented comprehensive approaches to identify highly effective biomarkers of irAEs from becoming standard practice [14–17]. Accumulating evidence provided insights that the gut microbiota could be leveraged for mounting antitumor efficacy with ICI therapy which might also influence the development of irAEs [18–25]. The baseline gut microbiota composition could serve as an important determinant of irAE onset [23, 25]. However, the present microbial traits on the baseline for discriminating susceptible optimates were inconsistent among studies [19], probably accounted for by the limited study size and discrepant treatment strategy. Accordingly, integrated analysis with more microbiome data was required for identifying effective microbial biomarkers for toxicity prediction.

Recent studies have also proposed gut microbiome-based predictive models for ICI efficacy of specific diseases [26–29]. However, highly effective microbial biomarkers for predicting the development of irAEs using more pooled data have been hardly proposed and are under investigation [13]. Developing potential microbial biomarkers for predicting the irAE occurrence probabilities and discovering potential mechanisms driving checkpoint blockade toxicities will have important clinical implications [6]. More specific therapeutic strategies could be available for identifying high-risk patients prior to treatment and thus supplying preventive methods to mitigate adverse effects.

Herein, a machine-learning algorithm was employed to uncover potentially predictive gut microbiota associated with irAEs, particularly in patients undergoing treatment with anti-PD-1/PD-L1 drugs. The composition of the fourteen gut microbes at the treatment baseline presents an intriguing and novel investigational avenue for predicting the incidence of irAEs, supported by a robustly validated model performance. Furthermore, our study provides new insights into preventing irAEs and uncovers potential mechanisms from a microbiological functional perspective.

## Methods

### Public datasets retrieval and irAEs status definition

We extracted data from studies published on https://pubmed.ncbi.nlm.nih.gov/ that included 16S rRNA sequencing data from patients with information on irAEs results and the gut microbiome at the treatment baseline. The data specifically encompassed pre-treatment or initial on-treatment samples as identified in the original papers, representing the gut microbiome at the treatment baseline. All sample identifiers and available metadata are provided in the Additional file 1: Table S1. Raw sequencing data of these studies were downloaded using the SRA toolkit (V.2.9.1) from Sequence Read Archive (SRA) using the following identifiers: PRJNA665109 (https://www.ncbi.nlm.nih.gov/bioproject/PRJNA665109) for Cascone et al. [30], PRJNA687361 (https://www.ncbi.nlm.nih.gov/bioproject/PRJNA687361) for Chau et al. [31], PRJNA606061 (https://www.ncbi.nlm.nih.gov/bioproject/PRJNA606061) for Hakozaki et al. [32], PRJEB48780 (https://www.ncbi.nlm.nih.gov/bioproject/PRJEB48780) for Zhang et al. [33], PRJNA762360 (https://www.ncbi.nlm.nih.gov/bioproject/PRJNA762360) for McCulloch et al. [20], PRJNA379764 (https://www.ncbi.nlm.nih.gov/bioproject/PRJNA379764) for Chaput et al. [34], and PRJNA302832 (https://www.ncbi.nlm.nih.gov/bioproject/PRJNA302832) for Dubin et al. [35]. A summary outlining the clinical characteristics of the studies under review is provided in Additional file 1: Table S2. The above studies were selected on the basis of the availability of sequencing data in public databases at the time when this study was initiated (Accessed Aug. 2022).

For the further meta-analysis, only three studies on a larger scale with more than 50 patients using anti-PD-1/PD-L1 drugs were included as the training datasets for model construction. Notably, we obtained the metadata of Hakozaki et al. [32] from the supplementary materials of another meta-analysis carried out by Shaikh et al. [36]. Only the baseline gut microbiome data defined by the original paper were included for the following analysis, and notably McCulloch et al. used a landmark timepoint to identify the baseline gut microbiome [20]. The irAEs status was evaluated using the original metadata and dichotomized to irAEs and non-irAEs, and irAEs were defined once the patients were recorded with any irAEs in the original paper and vice versa. Colon RNA sequencing data and 16S rRNA amplicon sequencing data from an independent cohort carried out by Baruch et al. [37] with accession numbers GSE162436 (https://www.ncbi.nlm.nih.gov/bioproject/?term=GSE162436) and PRJNA678737 (https://www.ncbi.nlm.nih.gov/bioproject/PRJNA678737) were utilized to speculate the potential mechanism for microbial traits of the model [37].

### Patient recruitment and sample collection

Baseline stool samples were collected from patients with pan-cancer who were initiated with anti-PD-1/PD-L1 drugs from Renji Hospital, Shanghai Jiao Tong University School of Medicine (SH Cohort, *N* = 65) and Xuzhou Central Hospital (JS Cohort, *N* = 50) (Table 1, Additional file 1: Table S3). Patient recruitment and sample collection were approved by the Medical Ethics Committee of Renji Hospital, Shanghai Jiao Tong University School of Medicine (ID: LY2020-067-B) and Xuzhou Central Hospital of Xuzhou Medical University (ID:XZXY-LJ-20200110-090). Written informed consent was obtained from each participant. This study protocol is in accordance with the approved World Medical Association Declaration of Helsinki (2008) and the Belmont Report. Treatment responses were evaluated through the Response Evaluation Criteria in Solid Tumors 1.1 (RECIST 1.1) [38] as determined in the original studies. Patients with complete response, partial response, or/and stable disease lasting more than 6 months, according to RECIST 1.1 criteria, were classified as responders, whereas patients with progressive disease or stable disease lasting less than 6 months were classified as non-responders. Immunotherapy-related adverse events were evaluated and identified retrospectively based on the Common Terminology Criteria for Adverse Events (CTCAE), version 5.0. Patients were recruited for initial diagnosis and had never received any ICI treatment before fecal sample collection. The stool was collected in fecal collection tubes and was stored at − 80 °C. We collected fresh blood samples from two groups of patients: those with confirmed irAEs (*N* = 10) and those without irAEs (*N* = 10) who had undergone anti-PD-1/PD-L1 blockade therapy. Serum was extracted by centrifugation at 3000 rpm for 10 min and stored at − 80 °C.

**Table 1:** Clinical features of the studies employed in this investigation

Study	Tumor type	Treatment	Group		BMI(%)	Sex F(%)/M(%)
Chau et al. [31]^a^	Lung cancer	anti-PD1	irAEs (*n* = 16); non-irAEs (*n* = 12)	-	-	-
Hakozaki et al. [32]^a,b^	Lung cancer	anti-PD1/PD-L1	irAEs (*n* = 16); non-irAEs (*n* = 54)	69.71 ± 9.58	-	42.3/57.7
Zhang et al. [33]^a,b^	Lung cancer	anti-PD1/PD-L1	irAEs (*n* = 26); non-irAEs (*n* = 43)	65.61 ± 9.56	< 25(47.1); ≥ 25(45.7); NE(7.1)	29.0/71.0
McCulloch et al. [20]^a,b^	Melanoma	anti-PD-1	irAEs (*n* = 35); non-irAEs (*n* = 16)	67.36 ± 12.48	< 25(19.6); ≥ 25(80.4)	35.3/64.7
Chaput et al. [34]^a^	Melanoma	anti-CTLA-4	irAEs (*n* = 7); non-irAEs (*n* = 19)	-	-	-
Dubin et al.^a^	Melanoma	anti-CTLA-4	irAEs (*n* = 10); non-irAEs (*n* = 24)	-	-	-
Cascone et al.	Lung cancer	Combined	irAEs (*n* = 20); non-irAEs (*n* = 19)	-	-	-
SH Cohort (in-house)^c^	Multi-type	anti-PD1/PD-L1	irAEs (*n* = 23); non-irAEs (*n* = 42)	63.57 ± 12.07	> 25 (15.4); ≤ 25(84.6)	26.2/73.8
JS Cohort (in-house)^c^	Multi-type	anti-PD1/PD-L1	irAEs (*n* = 16); non-irAEs (*n* = 34)	61.94 ± 13.17	> 25(20); ≤ 25(78); NE(2)	22.0/78.0

*Note*: Each study is indicated by first author and year of publication. ^a^Studies utilized for reanalyzing the differential microbes between irAEs and non-irAEs. ^b^Studies utilized for constructing predictive microbial Random Forest classifier for anti-PD-1/PD-L1-associated irAEs. ^c^Studies utilized as external validation. *PD-1* programmed death 1, *PD-L1* programmed death ligand 1, *CTLA-4* cytotoxic T lymphocyte-associated antigen 4, *NE/-* not evaluated/not available

### Data preprocessing

The 16S rRNA sequencing data were analyzed using QIIME2 (V.2022.2) [39], a plugin-based platform for microbiome analysis. DADA2 (V.2022.1) [40] software, wrapped in QIIME2, was used to filter out sequencing reads with quality score Q > 25 and denoise reads into ASVs, resulting in feature tables and representative sequences. Taxonomy classification was assigned based on the naïve Bayes classifier using the classify-sklearn package against the RDP Classifier_16S_V18 reference sequences [41]. ASVs that could not be precisely annotated to species were reassigned to ones having the most similar sequences in the same genus (or family) using NCBI Blast. Subsequently, representative sequences were aligned and merged within the same annotation on species using the function tax_glom() in phyloseq R package. Then, the feature tables were converted to relative abundance tables.

Raw FASTQ reads of shotgun metagenomic data underwent quality filtering using fastp with default parameters. Using mOTUs3 (Marker gene-based operational taxonomic units) [42] with default parameters, we profiled the microbial composition for each sample and counted the number of reads mapping to given phylogenetic genes. Each marker gene is given a specific operational taxonomic unit (OTU) acting as resolutive microbes. The output files were loaded into R and packaged into a phyloseq object for ease of analysis. The relative abundance values were calculated and converted for the following analysis.

The raw count data for transcriptomic analyses on normal colon tissue [37] were downloaded from GEO database repository (https://ftp.ncbi.nlm.nih.gov/geo/series/GSE162nnn/GSE162436/suppl/GSE162436_stranded_rev_CPM2_gut_TMM_counts.xlsx). Output files were further analyzed with R4.1.2 software. Differentially expressed genes were filtered by calculating the false discovery rate less than 0.5 (FDR < 0. 5) using the DESeq2 package [43] and visualized using ggplot2 in R. The ClusterProfiler package was utilized for performing Gene Set Enrichment Analysis (GSEA). Differential pathways from the Kyoto Encyclopedia of Genes and Genomes (KEGG) database with FDR less than 0.05 were finally visualized.

### Confounder analysis and covariate evaluation

We employed an ANOVA-like analysis referring to a previous study [27] to assess the impact of potential confounding variables and the presence of a disease. The total variance of a specific amplicon sequence variant (ASV) was compared to the variance explained by irAEs status (irAEs and non-irAEs) and the variance by confounding factors (age, BMI, sex, antibiotics, drug types, tumor staging, patients PD-L1 status, and study). Variance calculations were performed on ranks to account for non-Gaussian distribution of microbiome abundance data. Potential confounding factors with continuous values were transformed into discrete variables either as quartiles or in the case of BMI as groups of lean (> 25), overweight (25–30), and obese (> 30) based on conventional cutoffs, PD-L1 status were transformed into discrete variables with the cutoff value of 50%. To assess whether different tumor types influence the relationship between gut bacteria and irAEs, we employed a method known as aPCoA (covariate-adjusted principal coordinates analysis) [44].

### Meta-analysis of important abundant species

Considering the “study” accounted for the largest confounder, we used the MMUPHin (Meta-analysis Methods with a Uniform Pipeline for Heterogeneity in microbiome studies) [45] for microbial community batch correction on the confounder factor “study,” a new algorithm extending the batch correction method developed for gene expression data in ComBat with an additional component to allow for the zero-inflated nature of microbial abundance data. The significance of differential abundance was tested on a single ASV using a two-sided blocked Wilcoxon rank-sum test implemented in the R (V.4.1.2) “coin” package (To identify more potential microbial biomarkers, *P* values < 0.1 were considered to bring into the further analysis). Confounder with a high variance explanation was also defined as a block to adjust the batch effects in the differential analysis.

### Model construction and feature selection

The integrated anti-PD-1/PD-L1 cohort was utilized as the training subsample (*n* = 190), for which we developed the prediction algorithm. The features used for model building consist of important differential features (*P* < 0.1) as well as patient metadata features including age and sex. For the sake of incomplete data on the BMI, tumor stage, etc., we did not integrate other metadata for model construction.

Subsequently, the Random Forest (RF) models were built with 501 estimator trees and each tree had 10% of the total features. Then, an Iterative Feature Elimination (IFE) step was used to filter features and optimize the performance of subsequent RF models. The top features from the top-performing model were selected as “important features”. The permutation-based importance (function Permutation Importance) from the ELI5 Python package (https://eli5.readthedocs.io.) was finally utilized to compute the feature importance for models.

We used abundance profiles including the most important abundant microbial species and assessed how well classifiers trained in cross-validation on one study generalize in evaluations on the other two studies (termed as study-to-study transfer of classifier). And we also further assessed if including data from all but one study in model training improves prediction on the remaining hold-out study (also termed as leave-one-study-out (LOSO) validation).

### Model evaluation and external validation

Using the important microbiome features, we built RF Classifiers in the scikit-learn (V.0.19.2) package with stratified tenfold cross-validation to distinguish the patients with irAEs or non-irAEs. The receiver operating characteristic (ROC) curves and the area under the curve (AUC) were performed for model performance evaluation. To calculate the probability of irAEs onsets, we developed a robust score for the RF classifier with input microbial features (hereafter called RF score). The RF score for both the training and the validation datasets were calculated using function predict_proba() in the scikit-learn (V.0.19.2) package. The optimal thresholds of the RF score, which discriminate irAEs and non-irAEs, were computed by using Youden’s index method in the training set with the pROC package. The higher RF score indicated a lower probability for patients developing irAEs. Model predictive performance was measured by multi-metrics including sensitivity, specificity, accuracy, positive predictive value (PPV), and negative predictive value (NPV). Model specificity evaluation was conducted between irAEs and response using the Bootstrap Hypothesis testing.

Two additional cohorts, including the Shanghai Cohort (SH cohort, *N* = 65) and Jiangsu Cohort (JS cohort, *N* = 50) were used as independent cohorts for validation. The clinical information is demonstrated in Additional file 1: Table S3. RF scores for the validation datasets were calculated using the function predict_proba () in the scikit-learn (V.0.19.2) package. Then ROC curves and the AUC value for model performance evaluation were conducted using the pROC function.

### Functional profile analysis

To investigate the potential mechanisms of the functions of our model species, PICRUSt2 (V.2.0.3) as previously published was utilized for predicting microbiome genome and function inferred from 16S rRNA sequences [46]. We collected the output results of PICRUSt2 predictions based on several gene family databases by default, including KEGG orthologs (KOs), Enzyme Commission numbers (EC numbers), and MetaCyc pathway.

The HMP Unified Metabolic Analysis Network 2 (HUMAnN2) tool [47] was further utilized for profiling the microbial function from shotgun metagenomic data. Genes/proteins and pathways abundances of the microbiota between samples were quantified using UniRef50 gene clusters in conjunction with MetaCyc databases.

### DNA extraction, 16S rRNA gene sequencing, and shotgun metagenome sequencing

DNA was extracted from fecal samples using HiPure Stool DNA Mini Kit (Magen, D314103, China) following the manufacturer’s instructions. The full-length primer sequences designed for amplifying the V3-V4 hypervariable region of 16S rRNA gene including Illumina adaptors were as follows: forward: CCTACGGGNGGCWGCAG, and reverse: GACTACHVGGGTATCTAATCC. Microbial genomic DNA was used to start the polymerase chain reaction (PCR) protocol. A volume of 1 μL of the PCR product was quantified using a fluorometer (Qubit, Invitrogen). After size verification, the libraries were sequenced using a 2 × 250 pb paired-end run on a MiSeq sequencer, according to the manufacturer’s instructions (Illumina).

DNA for shotgun metagenome sequencing was extracted and quantified as above. DNA was then sheared to the desired insert size, and products were brought to 50 μL using 1X VAHTS DNA Clean Beads. After end-repreparation and adapter ligation, libraries are generated and run through 10–15 cycles of PCR with KAPA Hyper Prep Kit (Roche Sequencing Solutions, Pleasanton, CA). The Qubit Fluorometer and the Agilent 2100 Bioanalyzer were used for library quantification. Finally, libraries were sequenced on the Illumina Nova6000 on a 2 × 150 bp paired-end run.

### qRT-PCR validation

Real-time qPCR to quantify the abundance and expression of the differential gene *menH* and *menC *was performed on a subset of samples in the Jiangsu Cohort (17non-irAEs and 11 irAEs). To quantify *menC* and *menH* genes relative to the total bacterial RNA/DNA in a sample, qPCR was performed in triplicates for the 16S rRNA and *menC* and *menH* genes, respectively. We utilized primers based on a previous study [29] for *menC* and *menH*, along with standard primers for universal eubacteria 16S as in prior research [48]. Real-time PCR reactions were prepared with a final primer concentration of 0.5 μM in a 20-μl final reaction volume and then were performed with a SYBR Green qPCR Mix on a StepOnePlus real-time PCR system (Applied Biosystems). Cycling conditions were performed as described in the protocol. − ΔCt values were calculated as the difference between *menC* or *menH* and 16S Ct values. The significance of the comparison between irAEs and non-irAEs samples was tested on the − ΔCt values using the Wilcoxon test as confirmation of metagenomic enrichment.

### Bacteria culture and supernatant collection

*Parabacteroides merdae* (*PM*, BNCC358131) and *Lactobacillus Salivarius* (*LS*, BNCC194719) were purchased from BeNa Culture Collection (BNCC, Shanghai, China). *PM* was cultured overnight at 37 °C under anaerobic conditions (DG250, Don Whitley Scientific, West Yorkshire, UK) in brain heart infusion (BHI) broth with the protocol. The *LS* was cultured in deMan Rogosa Sharpe (MRS) medium overnight at 37 °C in shake cultivation at 220 rpm/min. Bacteria were cultured until OD_600_1.0–1.2 and cultures were collected and stored as 0.5–1-ml aliquots at − 80 °C until used for the quantification of menaquinone.

### High-performance liquid chromatography analysis

The HPLC–MS/MS analysis followed the methodology described in previous studies [49, 50]. Separation was accomplished on the Zorbax Eclipse Plus C18 column (2.1 × 50 mm, 1.8 µm, 600 bar). Elution was carried out with the following solvent system: 0.1% formic acid in water (Mobile Phase A) and acetonitrile plus 0.1% formic acid (Mobile Phase B). The flow rate was set at 0.5 ml/min, employing an isocratic elution program with Pump A at 0% and Pump B at 100% for 10 min. Sample injection was carried out using an automatic sampler with a sample tray temperature maintained at 4 °C, and 20 μL of the sample was utilized for analysis. For quantifying menaquinone, Menaquinone-6 (MK-6) (Macklin, CAS #84–81-1) was employed as the standard.

## Results

### Characteristics of studies involved in analyzing irAEs-associated gut microbiomes

**Fig. 1:**
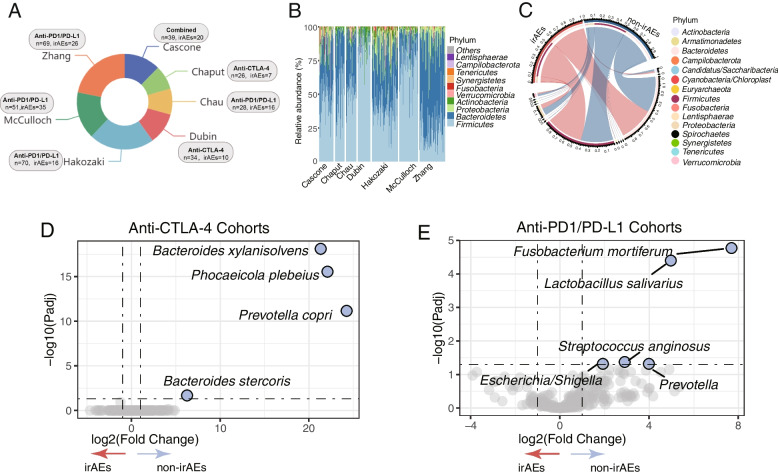
Study characteristics and microbiota composition difference in different irAEs status. **A** Study characteristics and demographic proportion of irAEs. **B** Microbiota composition (Phylum level) in the different studies of individuals. **C** Microbiota composition (Phylum level) in the different irAEs status analyzed with all cohorts demonstrated in 1A. **D** Visualization of differential microbiota in anti-CTLA-4 cohorts (Dubin and Chaput) by volcano plot. **E** Visualization of differential microbiota in anti-PD-1/PD-L1 cohorts (Zhang, Hakozaki, Chau, and McCulloch) by volcano plot

Based on the availability of baseline16S rRNA sequencing data and detailed population clinical information, we finally included seven studies for the following analysis (Table 1). Totally, we collected 218 patients receiving anti-PD-1/PD-L1 immunotherapy from 4 studies, 60 patients receiving anti-CTLA-4 immunotherapy from 2 studies, and 39 patients receiving combined anti-PD-1/PD-L1 and anti-CTLA-4 immunotherapy (Fig. 1A). We also observed there is no statistically significant association between adverse reaction outcomes and tumor types (*P*-value = 0.1523, Additional file 2: Fig.S1B). Subsequently, we waived the studies conducted by Chau et al. for the anti-PD-1/PD-L1-associated irAEs model construction for the sake of its small scale (*n* = 23). The detailed demographic information and study characteristics are demonstrated in Table 1 and Additional file 1: Table.S2. Similar to other classic sequencing data from human gut microbiota [51], *Firmicutes* and *Bacteroidetes* represented the two most dominant bacterial phyla of the total community among all studies. However, the relative abundance of microbiomes in the phylum level seemed to vary from different studies and irAEs status. Previous studies reported an elevated presence of *Firmicutes* was linked to a greater likelihood of experiencing irAEs, while the phyla *Bacteroidetes* and *Proteobacteria* tended to be more abundant in individuals without irAEs [25, 52]. In this study, despite no significant statistical difference, we found that *Firmicutes* appeared to be higher in patients with irAEs (median value, 0.316 vs. 0.273) while Bacteroidetes seemed to be lower in patients with irAEs (median value, 0.151 vs. 0.229) as compared to non-irAEs (Fig. 1B and C).

### Compositional differences in the microbiome between irAEs and non-irAEs under different ICIs

Several lines of evidence support the hypothesis that anti-CTLA-4 and anti-PD-1/PD-L1 therapy are associated with distinct microbial biomarkers [15, 53]. Hence, we tried to figure out differential gut microbiome for distinguishing irAEs and non-irAEs with anti-PD-1/PD-L1 and anti-CTLA-4 treatments, respectively. Several species, such as *Bacteroides xylanisolvens*, *Phocaeicola plebeius* and *Prevotella copri*, were found to enrich in non-irAEs in the pooled anti-CTLA-4 cohorts (Fig. 1D, Additional file 1: Table.S4). Strikingly, the integrated anti-PD-1/PD-L1 datasets demonstrated quite different species for distinguishing irAEs and non-irAEs (Fig. 1E, Additional file 1: Table.S5). The abundance of species including *Fusobacterium mortiferum* and *Lactobacillus salivarius* was significantly enriched in non-irAEs (Fig. 1E).

### Potential confounders adjustment and baseline gut microbial composition evaluation for distinguishing anti-PD-1/PD-L1 immunotherapy-associated irAEs and non-irAEs

**Fig. 2:**
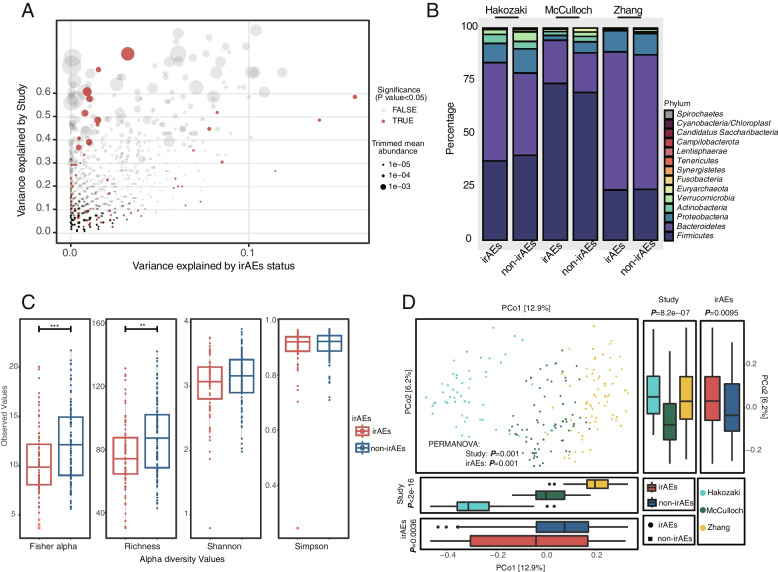
Alterations of gut microbial composition with different irAEs status among three anti-PD-1/PD-L1 cohorts. **A** Variance explained by irAEs status (irAEs versus non-irAEs) is plotted against variance explained by study effects for individual ASVs. The significantly differential ASVs are colored in red and the dot size is proportional to the abundance of each ASV. *P* values were from a two-sided blocked Wilcoxon rank-sum test. **B** Relative abundance of bacterial phyla in irAEs and non-irAEs across all three different studies. **C** Alpha diversity analysis calculated with Fisher alpha, Richness, Shannon, and Simpson indexes. **D** Principal coordinate analysis of samples (irAEs: *n* = 77; non-irAEs: *n* = 113) from all three anti-PD-1 studies based on Bray–Curtis distance. *P* values of beta diversity based on Bray–Curtis distance were calculated with PERMANOVA. The study is color-coded and the group (irAEs and non-irAEs) is indicated by different shapes. The upper-right and the bottom-left boxplots illustrate that samples projected onto the first two principal coordinates broken down by study and disease status, respectively. *P* values of the first and second principal components were calculated with a two-sided Kruskal–Wallis test for study and group. All boxplots represent the 25th–75th percentile of the distribution; the median is shown in a thick line at the middle of the box; the whiskers extend up to values within 1.5 times of IQR, and outliers are represented as dots. The anti-PD1/PD-L1 cohorts comprising over 50 patients, such as those led by Zhang et al., Hakozaki et al., and McCulloch et al., were utilized for the analysis in this part

To create a gut microbiome signature for predicting the occurrence of irAEs, we finally incorporated three studies with anti-PD-1/PD-L1 immunotherapy for the meta-analysis in consideration of the integrity of demographic information and sample size. Since both technical and biological confounders might exist in different studies, we calculated the variances explained by irAEs status and other clinical variates for each species to quantify the potential confounder effects (See Supplementary Materials and Methods). Remarkably, the variance of species explained by “Study” was found to be more predominant than other confounders (Fig. 2A, Additional file 2: Fig.S2). On the phylum level, *Firmicutes* and *Bacteroidetes*, accounting for the top two of the most predominant phyla, showed more variation in their ratios among studies (Fg.2B). Zhang et al.’s study demonstrated a higher level of *Bacteroidetes* while *Firmicutes* is mostly composed in the other two studies. Additionally, both the alpha diversity and beta diversity varied among different studies (*P* < 0.001, Fig. 2D, and Additional file 2: Fig.S1.C-D). All above indicated that the factor “study” brought a great impact on gut microbial composition. Therefore, we treated “study” as a blocking factor for the adjustment of the batch effect in the further analysis and a two-sided blocked Wilcoxon rank-sum test was utilized to test the significance between non-irAEs and irAEs.

The baseline gut microbiome of irAEs was demonstrated to be distinct from non-irAEs. The alpha diversity indexes including Fisher alpha and Richness were significantly higher in non-irAEs (*P* < 0.01, Fig. 2C). The Shannon index and Simpson index had also a higher trend in the non-irAEs group. In addition, the beta diversity calculated using *Bray–Curtis* distance was found to be significantly different in irAEs and non-irAEs after pooling the data sets (*P* = 0.001, PERMANOVA, Fig. 2D), whereas no significant differences were found in other clinical factors such as age and gender (*P* > 0.05, PERMANOVA, Additional file 2: Fig.S2B-C). This indicated the baseline gut microbiome constitutes were different in irAEs and non-irAEs. Convincingly, a set of differential microbes more significantly explained by “irAEs status” could be identified.

To assess whether different tumor types influence the relationship between gut bacteria and irAEs, we initially presented the PCoA plot without considering tumor types. Subsequently, we applied the aPCoA algorithm to correct for tumor types as covariates. Our findings suggest that there was no substantial alteration in the PCoA plots following correction for tumor types. Furthermore, the extent of variation explained by the PCoA experienced only minimal changes before and after tumor type correction (Additional file 2: Fig.S1E-F). This indicated that gut microbes might associate with the irAEs regardless of tumor type.

### Identification of irAEs-specific species and model construction for anti-PD-1/PD-L1 immunotherapy-associated irAEs

**Fig. 3:**
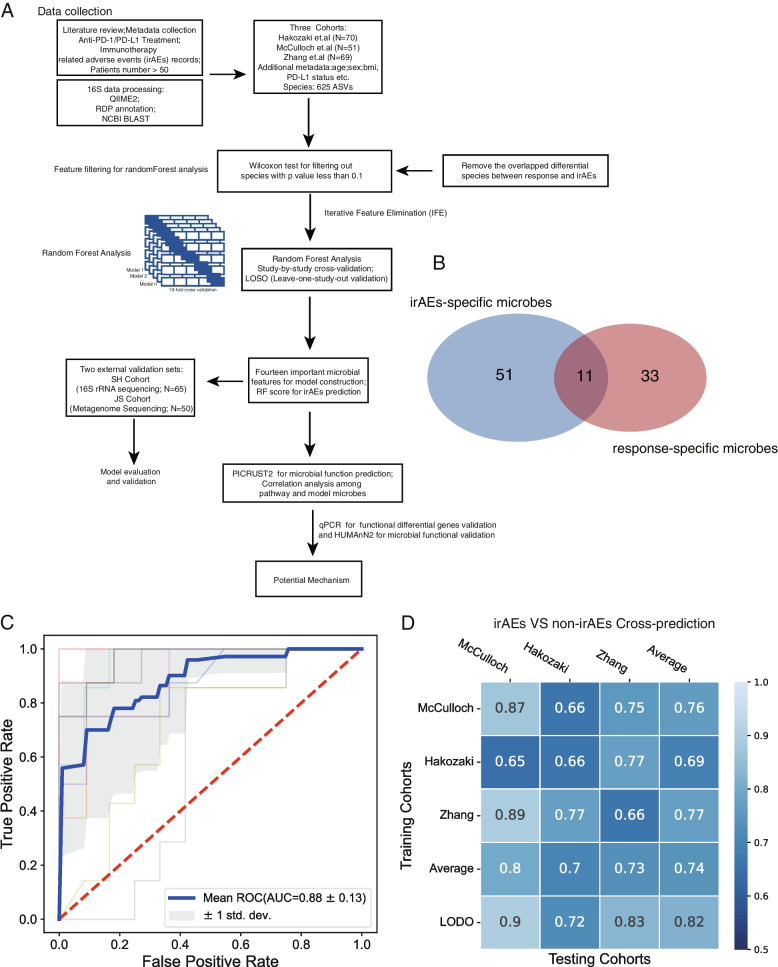
Model construction and performance. **A** Flow chart for microbial model construction. **B** Venn diagram shows the overlap of important microbiota assigned at species level between irAEs and response. **C** The AUC of the optimized models constructed with the most important microbiota for distinguishing non-irAEs from irAEs. Mean AUC and standard deviation of stratified tenfold cross-validation were shown. For each AUC detailed: “ROC Fold 1 (AUC = 0.89),” “ROC Fold 2 (AUC = 0.93),” “ROC Fold 3 (AUC = 0.97),” “ROC Fold 4 (AUC = 1.00),” “ROC Fold 5 (AUC = 0.94),” “ROC Fold 6 (AUC = 0.97),” “ROC Fold 7 (AUC = 0.91),” “ROC Fold 8 (AUC = 0.57),” “ROC Fold 9 (AUC = 0.71),” “ROC Fold 10 (AUC = 0.95).” **D** Prediction performance of important features using study-by-study and leave-one-study-out (LOSO) validation. The heatmap shows the area under the receiver operating characteristic curve (AUROC) from cross-validations within each study (blue boxes along the diagonal) and study-to-study model transfer (external-validations off-diagonal). The last column shows the average AUROC for study-to-study predictions. The bottom line shows the AUROC for a model trained on all studies but one (LOSO validation)

An outstanding question is to search for microbial biomarkers predicting anti-PD-1/PD-L1 immunotherapy-associated irAEs. The model construction pipeline is described in Fig. 3A. Sixty-two microbes were identified as irAEs-associated microbial traits, of which 11 species were also found to correlate with response. In order to construct more specific microbial traits model for irAEs, the intersection of response and irAEs-associated microbial traits were subsequently ruled out, and ultimately 51 important microbial features were regarded as irAE-specific species for the following feature filtering (Additional file 1: Table.S6). In total, 14 microbial features were filtered to show the best average AUC as well as the predominantly discriminatory power for identifying irAEs and non-irAEs after IFE (Additional file 2: Fig.S3A-O). A robust RF model was eventually constructed with a core set of best features, including 14 differential microbes (hereafter termed as RF14 classifier), which achieved an average AUC of 0.88 for distinguishing non-irAEs from irAEs (Fig. 3C, Additional file 1: Table.S7). Most of the species involved in the model were significantly enriched in the non-irAEs in the integrated data (Additional file 2: Fig.S3). Among these, the ASV assigned as *Lactobacillus salivarius* was identified as the top highest-ranking biomarker. To test whether the identified microbial features in our RF14 classifier are universal and robust across multiple studies, we performed study-to-study transfer validation and LOSO validation on the entire samples. In our RF14 classifier, the AUC values of study-to-study transfer validation ranged from 0.65 to 0.89, with an average of 0.74 (Fig. 3D). Additionally, the AUC values of LOSO analysis ranged from 0.72 to 0.90 (average AUC = 0.82, Fig. 3D), which was better than those achieved in study-to-study transfer validation owing to using a larger amount of training data (Fig. 3D). All above demonstrated that our microbial-derived biomarker panel had excellent accuracy across studies.

### Machine learning-based microbiome model performance assessment and external validation

**Fig. 4:**
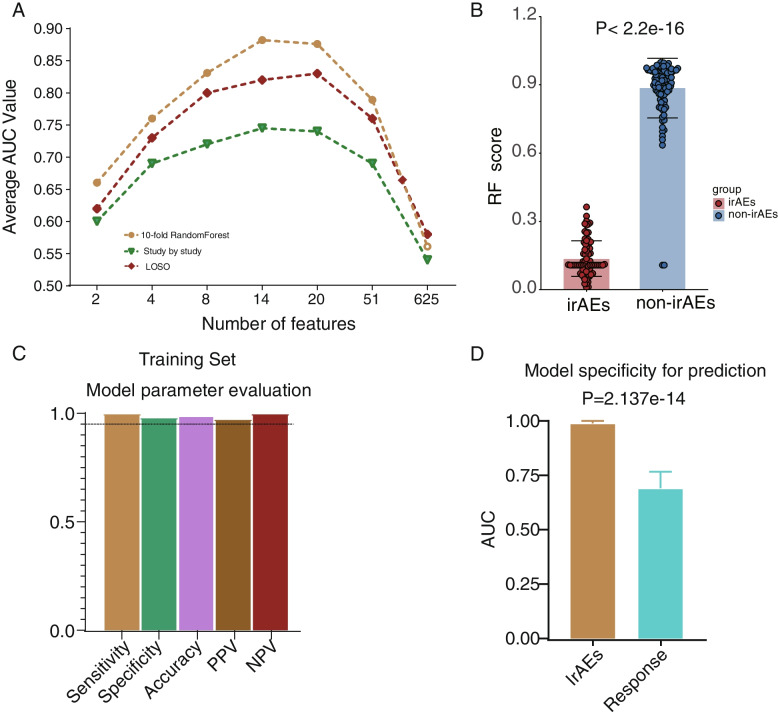
Identification of minimal features for the best model performance and multiple metrics for model evaluation. **A** Average AUC of ten-fold Random Forest cross-validation, study-to-study transfer validation classifiers, and LOSO validation for non-irAEs versus irAEs with a different number of features. **B** Comparison of RF score distributions calculated by RF14 classifier between non-irAEs (*n* = 113) and irAEs (*n* = 77) groups. Two-sided *P* values were calculated using the Wilcoxon rank-sum test. **C** Performance measurements of RF14 classifier illustrated by sensitivity, specificity, accuracy, positive predictive value (PPV), and negative predictive value (NPV). **D** The comparison of microbiome model specificity between irAEs and response. The anti-PD1/PD-L1 cohorts comprising over 50 patients, such as those led by Zhang et al., Hakozaki et al., and McCulloch et al., were utilized for the analysis in this part

We used a different number of input features, including all features, differential features, all important features, and top features (feature ranks after calculating the permutation-based importance), to test the predictive capability. The average AUC values were consistently calculated and compared in study-to-study transfer validation, LOSO validation, and the integrated ten-fold Random Forest analysis, respectively. Obviously, the set of 14 microbes achieved higher predictive performance compared to other number of features used in all evaluation methods (Fig. 4A). The RF score derived from our RF14 classifier for non-irAEs was significantly higher than irAEs (Fig. 4B, Wilcoxon rank-sum test). We further calculated the predictive performance of the gut microbiome-based model as measured by sensitivity, specificity, accuracy, positive predictive value (PPV), and negative predictive value (NPV). The RF14 classifier showed high predictive performance as measured by sensitivity, specificity, accuracy, PPV, and NPV (Fig. 4D and Additional file 2: Fig.S4A). Moreover, as expected, our RF14 classifier demonstrated significant specificity for predicting the occurrence of irAEs instead of ICIs efficacy (Fig. 4D, *P* = 2.137e − 14, Bootstrap Hypothesis Test).

**Fig. 5:**
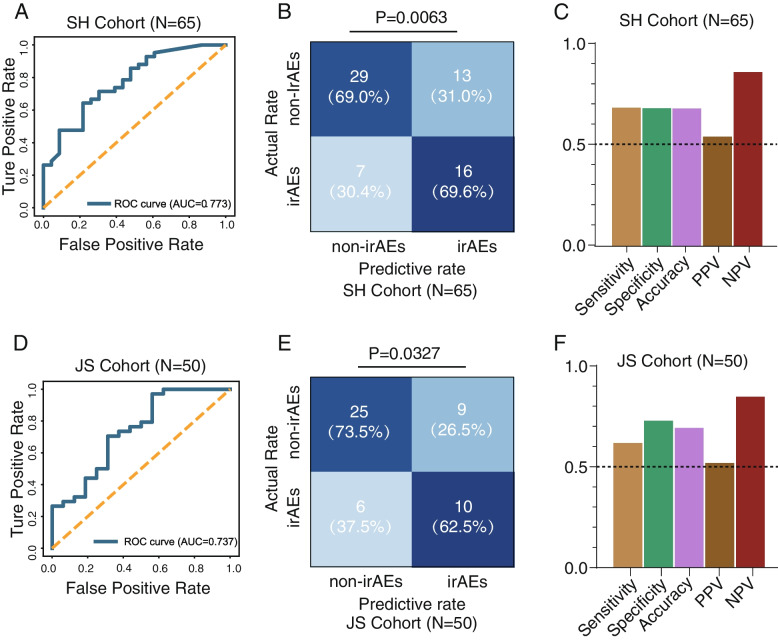
External validation cohorts and evaluation metrics for microbiome model. **A** Receiver operating characteristic (ROC) curves for the validation of microbiome model using SH amplicon cohort (*n* = 65). **B** Statistical analysis was conducted based on the predictive value and actual value of irAEs using the optimal thresholds of RF score defined in the training cohort from SH amplicon cohort (*n* = 65), chi-square test. **C** Performance measurements of RF14 classifier for SH cohort illustrated by sensitivity, specificity, accuracy, positive predictive value (PPV), and negative predictive value (NPV). **D** Receiver operating characteristic (ROC) curves for the validation of microbiome model using JS metagenome cohort (*n* = 50). **E** Statistical analysis was conducted based on the predictive value and actual value of irAEs using the cut-off value of RF score defined in the training cohort from JS metagenome cohort (*n* = 50), chi-square test. **F** Performance measurements of RF14 classifier for JS cohort illustrated by sensitivity, specificity, accuracy, positive predictive value (PPV), and negative predictive value (NPV)

To further validate our RF14 classifier for irAEs prediction, two additional independent cohorts, SH cohort (*N* = 65) and JS cohort (*N* = 50), were incorporated into this study. SH cohort consisted of 23 irAEs and 42 non-irAEs patients using 16S rRNA amplicon sequencing strategy for gut microbiome analysis. And JS cohort, consisting of 16 irAEs and 34 non-irAEs, adopted shotgun metagenome sequencing strategy. We calculated the relative RF score for the validation cohorts and used the precision-recall (PR) curves and AUC value under the ROC curve for model evaluation. Both the SH cohort and JS cohort achieved reasonable performance for distinguishing non-irAEs from irAEs, with relatively superior model metrics (Fig. 5A–F). This indicated that the gut microbiome identified in our RF14 classifier possessed robustness for discriminating non-irAEs from irAEs.

Meanwhile, using the same model construction methods, we reconstructed RF models for the small-scale dataset from Chau et al. and identified five important species achieving the best AUC value of 0.95 (Additional file 2: Fig.S4E). Notably, the important biomarkers were 3 out of 5 included in our discovery RF14 classifier. Additionally, the features’ ranks were consistent with our RF14 classifiers. For instance, ASVs assigned as *Lactobacillus salivarius* and *Parabacteroides goldsteinii* were also confirmed as the top-ranking biomarkers in our RF14 classifier (Additional file 1: Table.S7). Our research results indicate that the model constructed with features selected at* P* < 0.05 ultimately included 18 microbial features and achieved a maximum area under the curve (AUC) of 0.83 (Additional file 2: Fig.S4F).

### Microbial function exploration and validation

**Fig. 6:**
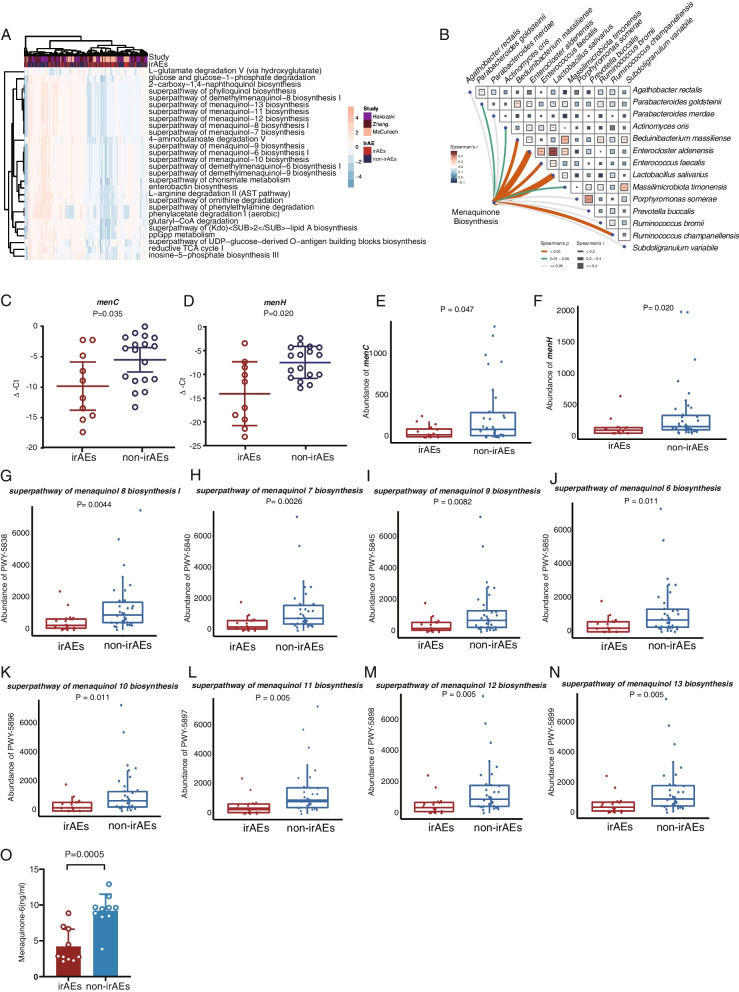
Microbial functional alterations in irAEs and non-irAEs. **A** Differentially abundant pathways were plotted; *P* values were computed using a two-sided blocked Wilcoxon rank-sum test and the FDR < 0.005 were presented in the heatmap. **B** The correlation between the abundance of menaquinone biosynthesis-related pathway and model species. Spearman’s correlation between the abundance of menaquinone biosynthesis and 14 representative microbial species in the classifiers and edge width corresponds to the Spearman’s *r* statistic and edge color denotes the statistical significance.* r*, Spearman correlation coefficient; with a color-gradient denoting Spearman’s correlation coefficients, and the exact values were described in heatmap frames. **C**, **D** Plotted values are qRT-PCR quantifications of bacterial genes in menaquinone biosynthesis. The abundances of *menC* (**C**) and *menH* (**D**) were compared between non-irAEs (*n* = 17) and irAEs (*n* = 11) groups. All boxes extend from 25 to 75th percentiles and whiskers show the minimum and maximum values. Lines at the middle of each box show the median. *P* values were computed using a two-sided Wilcoxon rank-sum test. **E**–**M** Shotgun metagenome functional validation (*N* = 50) for differential genes *menC* (**E**), *menH* (**F**), and menaquinone biosynthesis pathway (**G**–**M**). All boxes extend from 25 to 75th percentiles and whiskers show the minimum and maximum values. Lines at the middle of each box show the median. *P* values were computed using a two-sided Wilcoxon rank-sum test. **O** Blood concentration comparison of menaquinone-6 (MK-6) between patients with irAEs( *N* = 10) and patients without irAEs(*N* = 10). Statistical significance was assessed using a two-sided Wilcoxon rank-sum test

We further examined the microbiome-based functional alterations using the 16S rRNA data from training datasets. There were 110 differential pathways between irAEs and non-irAEs based on the results of MetaCyc pathway abundances calculated in PICRUSt2 (Additional file 1: Table.S8). Differential pathways with the FDR less than 0.005 were filtered out and clustered based on their abundance (Fig. 6A).

Notably, the menaquinone (also termed as vitamin K2) biosynthesis was significantly enriched in non-irAEs compared with irAEs (Fig. 6A, Additional file 1: Table.S8). The biosynthesis of 1,4-dihydroxy-2-naphthoate, an important intermediate of menaquinone biosynthesis, was also found to be enriched in non-irAEs (Additional file 1: Table.S8). We further calculated Spearman’s correlation between the menaquinone-related pathways and the abundance of species involved in our RF14 classifier. Interestingly, the menaquinone biosynthesis significantly correlated to the species that enriched in non-irAEs (Fig. 6B), which was also consistent when performing PICRUSt2 analysis on Chaput’s and Dubin’s cohorts (Additional file 2: Fig.S5 C-H). Moreover, 420 differential limited enzymes were identified from the EC metagenome prediction (Additional file 1: Table.S9). Consistently, we noticed that several rate-limiting enzymes in the biosynthesis of menaquinone, including *menH* (EC:4.2.99.20) and *menC* (EC:4.2.1.113), were significantly elevated in non-irAEs compared with that of irAEs in pooled data sets. These results were also confirmed by qPCR with our newly collected fecal samples (Fig. 6C, D), showing that *menH* and *menC* genes were significantly increased in the non-irAEs samples than those in the irAEs samples. Collectively, the declining menaquinone biosynthesis and its key genes from gut microbes were more related to the higher incidence of irAEs.

Since shotgun metagenomic sequencing data allow for a more accurate analysis of the microbial function, we further used additional shotgun metagenomics data derived from JS Cohort (*N* = 50) to examine the alteration of metabolic pathways and orthologous gene families between patients with irAEs and non-irAEs. Consistently, the expression of microbial genes *menH* and *menC* were significantly upregulated in non-irAEs (Fig. 6E, F). In addition, pathway analysis demonstrated the abundance of related pathways referring to the biosynthesis of menaquinone was significantly enriched in non-irAEs (Fig. 6G–N). All results indicated that microbiome-derived menaquinone might serve as potential functional microbial metabolites for defending the occurrence of irAEs.

### Quantification analysis on menaquinone with HPLC–MS

We performed HPLC–MS analysis of menaquinone in bacterial metabolites and serum samples collected from individuals both with and without irAEs. Notably, HPLC–MS/MS results confirmed menaquinone production in bacterial supernatants, particularly from two representative microorganisms, *Parabacteroides merdae* and *Lactobacillus salivarius*, when compared to control media (refer to Additional file 2: Fig.S5C-F). This highlights the potential of these species for menaquinone biosynthesis. Furthermore, serum menaquinone levels showed a significant increase in individuals without irAEs compared to those with irAEs (*P* = 0.005, as depicted in Fig. 6O).

### Potential mechanism deduction

To further elucidate how the microbial features involved in our RF14 classifier contribute to the development of irAEs, we used an extra study, consisting of 9 melanoma patients who received anti-PD-1/PD-L1 treatment with both fecal 16S rRNA amplicon sequence and bulk RNA sequencing on the normal intestinal or colorectal mucosa available. The nine patients were then stratified by the RF14 classifier into RF score-high and score-low groups via the median value. Notably, the inflammation-associated gene, *CCL21*, was significantly enriched in the RF score-low group (Additional file 1: Table.S10; Additional file 2: Fig. S5A). Interestingly, several pro-inflammation pathways including the chemokine signaling pathway and NF-κB signaling pathway were significantly elevated in the score-low group utilizing Gene Set Enrichment Analysis (GSEA) (Additional file 2: Fig.S5 B, Additional file 1: Table.S11). Remarkably, several reports had demonstrated that NF-κB signaling pathway could be inhibited by menaquinone, resulting in anti-inflammation effects [54, 55].

Taken together, these results indicated that the metabolites derived from the gut microbiome like menaquinone might exert potential protective effects via inhibiting the pro-inflammation signaling pathway such as NF-κB signaling pathway. Our data supported the idea that supplemented menaquinone during the anti-PD-1/PD-L1 immunotherapy might mitigate or prevent the occurrence of irAEs.

## Discussion

ICI drugs elicit the destruction of cancer cells by reliving inhibitory T cell signaling, while tipping the balance between the normal tissue and immune system as well. Predictive strategies that define the risk of irAEs are essential for optimizing ICI use, or alternatively for redirecting patients towards safer therapeutic modalities. Heretofore, growing evidence has demonstrated cytokines potentiate the development of irAEs, and cytokine-targeted therapies have been established for the long-term alleviation of irAEs and brought into clinical use for curbing severe irAEs [56]. However, the preventive strategy tailored for irAEs remains a challenge since “one size fits all” is not adequate for this setting. Gut microbiome and their metabolites have demonstrated effective synergistic antitumor response with ICI therapy and alleviate the toxicity induced by ICI drugs.

Data from the preclinical models and clinical observations indicated that the gut microbiome processed great potential in modulating the development of irAEs [21]. Pdcd1 ^− / − ^mice, which lack the inhibitory receptor Pdcd-1 and are usually utilized as a model mimicking the systematic function of anti-PD-1 drugs [57, 58], demonstrated obvious composition shift and diversity reduction on the gut microbiota, especially a dramatic reduction of *Lactobacillales* [59]. This was consistent with the microbial traits identified in our model, where we found the top-ranking species *Lactobacillus salivarius* for predicting irAEs, and exerts the strongest relevance with menaquinone abundance. In addition, exogenous supplements of probiotics such as *Bifidobacterium* and *Lactobacillus* were also confirmed to ameliorate the DSS plus anti-CTLA-4 colitis in murine model [60, 61]. *Parabacteroides* genus and *Ruminococcus* genus were also deciphered to be probiotics for the remission of inflammation and disease development [62]. Of note, two species of *Parabacteroides* genus (including *Parabacteroides goldsteinii* and *Parabacteroides merdae*), as well as two *Ruminocuccus* genera (*Ruminocuccus bromii* and *Ruminocuccus champanellensis*), were also identified as important microbial traits for predicting irAEs onset. Collectively, the microbial traits identified in our model might harbor promising function influencing the development of irAEs whereas more evidence was needed.

Overall, we utilized a robust machine-learning method to identify a panel of gut microbiome for predicting the occurrence irAEs from pooled data regardless of the various confounders. The baseline gut microbiome could function as effectively predictive biomarkers for irAEs with high sensitivity and specificity. External amplicon sequencing data and shotgun metagenome sequencing data were further carried out to validate the robust results. Additionally, from microbial functional analysis, in patients with irAEs, levels of menaquinone biosynthesis and the key enzymes involved in menaquinone synthesis are lower than in non-irAEs. This was similar to the previous findings on melanoma patients treated with anti-CTLA-4 inhibitors, where bacterial metabolism pathways involved in B vitamin biosynthesis were indicated to protect patients from immune-related colitis [35]. Based on the above findings, we deduced that regulation of the gut microbiome or the use of gut microbiome-derived metabolites like menaquinone, either prophylactically or concurrently with anti-PD-1/PD-L1 treatment, might harbor the potential to control and prevent the incidence of irAEs. It remains unclear whether reduced menaquinone levels contribute to irAEs onsets or development and further research was in demand.

Moreover, integrated analysis of the gut microbiome and colon tissue bulk RNA sequencing further showcased the potential mechanism for the inhibition of the NF-κB signaling pathway. Similarly, Luoma et al. [63] delineated a significantly elevated TNFα signaling via NF-κB in the colonic macrophage through a comprehensive single-cell analysis of immune cell population in patients with ICIs-associated colitis. The gut microbiome and its metabolites might interact with the host genome and restore the host immune cells, causing inflammation activation. Accordingly, more rationales were required to interpret how gut microbiome influenced host and immune cells.

Some controversy remains to be clarified regarding whether the treatment of irAEs might disrupt the efficacy of immunotherapy. For instance, non-small cell lung cancer patients who develop G3 or G4 pneumonitis and consequently receive high-dose corticosteroids for at least 4–6 weeks tend to have a worse prognosis. Conversely, patients with melanoma who discontinued treatment due to irAEs and utilized immune suppressant agents did not exhibit distinct outcome [64–66]. With the identification of those predictive biomarkers of irAEs, including gut microbiota biomarkers, it becomes possible to greatly enhance efficacy while mitigating irAEs [67, 68].

Our study also has certain limitations, primarily related to the study’s size and various factors confounding the omics data used for analysis. To gain a more comprehensive understanding of the biological markers associated with irAEs, it is important to improve the experimental design by controlling unnecessary confounding factors for more relevant clinical data. This will enable us to delve deeper into the complex mechanisms underlying the gut microbiome and the occurrence of irAEs [69]. Furthermore, it is essential to bolster our findings with in vivo and in vitro experiments. The validation of menaquinone’s functionality could be further substantiated through additional preclinical modeling.

## Conclusion

Our study underscores the predictive potential of microbial biomarkers for irAE onset. Menaquinone, derived from the microbiome, emerges as a promising therapeutic agent to modulate irAE occurrence. 

### Supplementary Information

**Additional file 1: Table S1.** Sample information for downloaded raw data. **Table S2.** Clinical characteristics of reviewed studies. **Table S3.** Clinical characteristics of in-house cohorts. **Table S4.** Differential microbial species between irAEs and non-irAEs in the integrated anti-CTLA-4 datasets. **Table S5.** Differential microbial species between irAEs and non-irAEs in the integrated anti-PD-1/PD-L1 datasets. **Table S6.** Wilcox-rank sum test for species filtering between with blocking on the ‘Study’. Important species (all P value < 0.1) compared between irAEs and non-irAEs and between responders and non-responders, were showcased in the data sheet, respectively. **Table S7.** Fourteen Species and relative abundance for model classifier. **Table S8.** Wilcox-rank sum test for pathway from PICRUST2 output. Differential pathways (FDR < 0.005) were showcased in the data sheet. **Table S9.** Wilcox-rank sum test for EC number from PICRUST2 output. Differential EC (FDR < 0.005) were showcased in the data sheet. **Table S10.** Differential genes (FDR < 0.5) from the colon tissue RNA sequencing (N = 9) were showcased in the data sheet. **Table S11.** Differential KEGG pathways (FDR < 0.05) analyzed by gene set enrichment analysis (GSEA).

**Additional file 2: Fig S1.** Alpha and beta diversity analysis in each cohort. **Fig S2.** Confounder analysis for model construction. **Fig S3.** Wilcoxon rank-sum test for the relative abundance comparison within 14 model species. **Fig S4.** Model evaluation in machine learning. **Fig S5.** Integrated analysis with colon tissue RNA sequencing and 16S rRNA amplicon sequencing. **Fig S6.** Qualitative analysis on menaquinone in key microbes.

## Data Availability

All the datasets used in the study that you already listed in the “Methods” section. Additional files are provided with this paper. All sequencing microbiome data that support the findings have been deposited at https://ngdc.cncb.ac.cn/gsa. Raw data on amplicon sequencing are accessible via BioProject accession number CRA014186 (https://ngdc.cncb.ac.cn/gsa/browse/CRA014186) [70], and raw data are available via BioProject accession number CRA014185 (https://ngdc.cncb.ac.cn//gsa/browse/CRA014185) [71] and CRA013442 (https://ngdc.cncb.ac.cn//gsa/browse/CRA013442) [72] for metagenome sequencing. And the source code is available at GitHub (https://github.com/mnhu-work/IrAE_microbiome) [73].

## Abbreviations

AUC: Average area under the receiver operating characteristic curve
CTLA-4: Cytotoxic T lymphocyte-associated antigen 4
CTCAE: Common Terminology Criteria for Adverse Events
HUMAnN2: HMP Unified Metabolic Analysis Network 2
IFE: Iterative Feature Elimination
irAEs: Immune adverse-related events
GSEA: Gene Set Enrichment Analysis
KEGG: Kyoto Encyclopedia of Genes and Genomes
MMUPHin: Meta-analysis Methods with a Uniform Pipeline for Heterogeneity in microbiome studies
NPV: Negative predictive value
OS: Overall survival
qPCR: Quantitative polymerase chain reaction
ICIs: Immune checkpoint inhibitors
PFS: Progression-free survival
PD-1: Programmed death 1
PD-L1: Programmed death ligand 1
FDR: False discovery rate
PPV: Positive predictive value
RF: Random Forest
RECIST 1.1: Response Evaluation Criteria in Solid Tumors 1.1
OTU: Operational taxonomic unit
SRA: Sequence Read Archive

## References

[1] Waldman AD, Fritz JM, Lenardo MJ (2020). A guide to cancer immunotherapy: from T cell basic science to clinical practice. Nat Rev Immunol.

[2] Couzin-Frankel J (2013). Breakthrough of the year 2013. Cancer immunotherapy. Science.

[3] Topalian SL (2012). Safety, activity, and immune correlates of anti-PD-1 antibody in cancer. N Engl J Med.

[4] Topalian SL, Drake CG, Pardoll DM (2015). Immune checkpoint blockade: a common denominator approach to cancer therapy. Cancer Cell.

[5] Baxi S (2018). Immune-related adverse events for anti-PD-1 and anti-PD-L1 drugs: systematic review and meta-analysis. BMJ.

[6] Dougan M, Luoma AM, Dougan SK, Wucherpfennig KW (2021). Understanding and treating the inflammatory adverse events of cancer immunotherapy. Cell.

[7] Okiyama N, Tanaka R (2022). Immune-related adverse events in various organs caused by immune checkpoint inhibitors. Allergol Int.

[8] Salem JE (2018). Cardiovascular toxicities associated with immune checkpoint inhibitors: an observational, retrospective, pharmacovigilance study. Lancet Oncol.

[9] Khoja L, Day D, Wei-Wu Chen T, Siu LL, Hansen AR (2017). Tumour- and class-specific patterns of immune-related adverse events of immune checkpoint inhibitors: a systematic review. Ann Oncol.

[10] Chen JH, Pezhouh MK, Lauwers GY, Masia R (2017). Histopathologic features of colitis due to immunotherapy with anti-PD-1 antibodies. Am J Surg Pathol.

[11] Das S, Johnson DB (2019). Immune-related adverse events and anti-tumor efficacy of immune checkpoint inhibitors. J Immunother Cancer.

[12] Abu-Sbeih H (2019). Immune checkpoint inhibitor-induced colitis as a predictor of survival in metastatic melanoma. Cancer Immunol Immunother.

[13] Morad G, Helmink BA, Sharma P, Wargo JA (2021). Hallmarks of response, resistance, and toxicity to immune checkpoint blockade. Cell.

[14] Xu Y, Fu Y, Zhu B, Wang J, Zhang B (2020). Predictive biomarkers of immune checkpoint inhibitors-related toxicities. Front Immunol.

[15] Sullivan RJ, Weber JS (2022). Immune-related toxicities of checkpoint inhibitors: mechanisms and mitigation strategies. Nat Rev Drug Discov.

[16] Johnson DB, Nebhan CA, Moslehi JJ, Balko JM (2022). Immune-checkpoint inhibitors: long-term implications of toxicity. Nat Rev Clin Oncol.

[17] Collins M (2017). Inflammatory gastrointestinal diseases associated with PD-1 blockade antibodies. Ann Oncol.

[18] Chang AE (2021). Targeting the gut microbiome to mitigate immunotherapy-induced colitis in cancer. Trends Cancer.

[19] Pezo RC, Wong M, Martin A (2019). Impact of the gut microbiota on immune checkpoint inhibitor-associated toxicities. Therap Adv Gastroenterol.

[20] McCulloch JA (2022). Intestinal microbiota signatures of clinical response and immune-related adverse events in melanoma patients treated with anti-PD-1. Nat Med.

[21] Andrews MC (2021). Gut microbiota signatures are associated with toxicity to combined CTLA-4 and PD-1 blockade. Nat Med.

[22] Naqash AR (2021). The role of gut microbiome in modulating response to immune checkpoint inhibitor therapy in cancer. Ann Transl Med.

[23] Lam KC, Goldszmid RS (2021). Can gut microbes predict efficacy and toxicity of combined immune checkpoint blockade?. Cancer Cell.

[24] Inamura K (2020). Roles of microbiota in response to cancer immunotherapy. Semin Cancer Biol.

[25] Wang Y, Jenq RR, Wargo JA, Watowich SS. Microbiome influencers of checkpoint blockade-associated toxicity. J Exp Med 2023;220. 10.1084/jem.20220948. 10.1084/jem.20220948 PMC9836236 36622383

[26] Su Q. et al. Faecal microbiome-based machine learning for multi-class disease diagnosis. Nature Communications 2022;13. 10.1038/s41467-022-34405-3 10.1038/s41467-022-34405-3 PMC9649010 36357393

[27] Wirbel J (2019). Meta-analysis of fecal metagenomes reveals global microbial signatures that are specific for colorectal cancer. Nat Med.

[28] Yachida S (2019). Metagenomic and metabolomic analyses reveal distinct stage-specific phenotypes of the gut microbiota in colorectal cancer. Nat Med.

[29] Wu Y (2021). Identification of microbial markers across populations in early detection of colorectal cancer. Nat Commun.

[30] Cascone T (2021). Neoadjuvant nivolumab or nivolumab plus ipilimumab in operable non-small cell lung cancer: the phase 2 randomized NEOSTAR trial. Nat Med.

[31] Chau J (2021). Prospective correlation between the patient microbiome with response to and development of immune-mediated adverse effects to immunotherapy in lung cancer. BMC Cancer.

[32] Hakozaki T (2020). The gut microbiome associates with immune checkpoint inhibition outcomes in patients with advanced non-small cell lung cancer. Cancer Immunol Res.

[33] Zhang F. et al. Analysis of the gut microbiota: an emerging source of biomarkers for immune checkpoint blockade therapy in non-small cell lung cancer. Cancers (Basel) 2021;13. 10.3390/cancers13112514 10.3390/cancers13112514 PMC8196639 34063829

[34] Chaput N (2017). Baseline gut microbiota predicts clinical response and colitis in metastatic melanoma patients treated with ipilimumab. Ann Oncol.

[35] Dubin K (2016). Intestinal microbiome analyses identify melanoma patients at risk for checkpoint-blockade-induced colitis. Nat Commun.

[36] Shaikh FY (2021). A uniform computational approach improved on existing pipelines to reveal microbiome biomarkers of nonresponse to immune checkpoint inhibitors. Clin Cancer Res.

[37] Baruch EN (2021). Fecal microbiota transplant promotes response in immunotherapy-refractory melanoma patients. Science.

[38] Eisenhauer EA (2009). New response evaluation criteria in solid tumours: revised RECIST guideline (version 1.1). Eur J Cancer.

[39] Bolyen E (2019). Reproducible, interactive, scalable and extensible microbiome data science using QIIME 2. Nat Biotechnol.

[40] Callahan BJ (2016). DADA2: High-resolution sample inference from Illumina amplicon data. Nat Methods.

[41] Cole JR (2009). The Ribosomal Database Project: improved alignments and new tools for rRNA analysis. Nucleic Acids Res.

[42] Ruscheweyh H-J. et al. 2022. 10.1101/2021.04.20.440600

[43] Love MI, Huber W, Anders S (2014). Moderated estimation of fold change and dispersion for RNA-seq data with DESeq2. Genome Biol.

[44] Shi Y, Zhang L, Do KA, Peterson CB, Jenq RR (2020). aPCoA: covariate adjusted principal coordinates analysis. Bioinformatics.

[45] Ma S (2022). Population structure discovery in meta-analyzed microbial communities and inflammatory bowel disease using MMUPHin. Genome Biol.

[46] Douglas GM (2020). PICRUSt2 for prediction of metagenome functions. Nat Biotechnol.

[47] Franzosa EA (2018). Species-level functional profiling of metagenomes and metatranscriptomes. Nat Methods.

[48] Kostic AD (2013). Fusobacterium nucleatum potentiates intestinal tumorigenesis and modulates the tumor-immune microenvironment. Cell Host Microbe.

[49] Manoury E, Jourdon K, Boyaval P, Fourcassié P (2013). Quantitative measurement of vitamin K2 (menaquinones) in various fermented dairy products using a reliable high-performance liquid chromatography method. J Dairy Sci.

[50] Ahmed S, Kishikawa N, Nakashima K, Kuroda N (2007). Determination of vitamin K homologues by high-performance liquid chromatography with on-line photoreactor and peroxyoxalate chemiluminescence detection. Anal Chim Acta.

[51] Qin J (2010). A human gut microbial gene catalogue established by metagenomic sequencing. Nature.

[52] Tan B (2022). Gut microbiota shed new light on the management of immune-related adverse events. Thorac Cancer.

[53] Wei SC (2019). Combination anti-CTLA-4 plus anti-PD-1 checkpoint blockade utilizes cellular mechanisms partially distinct from monotherapies. Proc Natl Acad Sci U S A.

[54] Ren L, Peng C, Hu X, Han Y, Huang H (2020). Microbial production of vitamin K2: current status and future prospects. Biotechnol Adv.

[55] Saputra WD, Aoyama N, Komai M, Shirakawa H. Menaquinone-4 suppresses lipopolysaccharide-induced inflammation in MG6 mouse microglia-derived cells by inhibiting the NF-kappaB signaling pathway. Int J Mol Sci 2019;20. 10.3390/ijms20092317. 10.3390/ijms20092317 PMC6540242 31083359

[56] Kang JH, Bluestone JA, Young A (2021). Predicting and preventing immune checkpoint inhibitor toxicity: targeting cytokines. Trends Immunol.

[57] Affolter T (2019). Inhibition of immune checkpoints PD-1, CTLA-4, and IDO1 coordinately induces immune-mediated liver injury in mice. PLoS ONE.

[58] Wei SC (2021). A genetic mouse model recapitulates immune checkpoint inhibitor-associated myocarditis and supports a mechanism-based therapeutic intervention. Cancer Discov.

[59] Wu D (2022). PD-1 signaling facilitates activation of lymphoid tissue inducer cells by restraining fatty acid oxidation. Nat Metab.

[60] Wang F, Yin Q, Chen L, Davis MM (2018). Bifidobacterium can mitigate intestinal immunopathology in the context of CTLA-4 blockade. Proc Natl Acad Sci U S A.

[61] Sun S (2020). Bifidobacterium alters the gut microbiota and modulates the functional metabolism of T regulatory cells in the context of immune checkpoint blockade. Proc Natl Acad Sci.

[62] Sasaki M (2022). The abundance of Ruminococcus bromii is associated with faecal butyrate levels and atopic dermatitis in infancy. Allergy.

[63] Luoma AM (2020). Molecular pathways of colon inflammation induced by cancer immunotherapy. Cell.

[64] Parakh S, Cebon J, Klein O (2018). Delayed autoimmune toxicity occurring several months after cessation of anti-PD-1 therapy. Oncologist.

[65] Horvat TZ (2015). Immune-related adverse events, need for systemic immunosuppression, and effects on survival and time to treatment failure in patients with melanoma treated with ipilimumab at Memorial Sloan Kettering Cancer Center. J Clin Oncol.

[66] Sznol M (2017). Pooled analysis safety profile of nivolumab and ipilimumab combination therapy in patients with advanced melanoma. J Clin Oncol.

[67] Lee SH (2021). Bifidobacterium bifidum strains synergize with immune checkpoint inhibitors to reduce tumour burden in mice. Nat Microbiol.

[68] Blum SM, Rouhani SJ, Sullivan RJ (2023). Effects of immune-related adverse events (irAEs) and their treatment on antitumor immune responses. Immunol Rev.

[69] Jing Y, Yang J, Johnson DB, Moslehi JJ, Han L (2022). Harnessing big data to characterize immune-related adverse events. Nat Rev Clin Oncol.

[70] Hu M. et al. Gut microbiome for predicting immune checkpoint blockade associated adverse events. CRA014186, Genome Sequence Archive, https://ngdc.cncb.ac.cn/gsa/browse/CRA014186. 10.1186/s13073-024-01285-9 PMC10799412 38243343

[71] Hu M. et al. Gut microbiome for predicting immune checkpoint blockade associated adverse events. CRA014185, Genome Sequence Archive, https://ngdc.cncb.ac.cn/gsa/browse/CRA014185. 10.1186/s13073-024-01285-9 PMC10799412 38243343

[72] Hu M. et al. Gut microbiome for predicting immune checkpoint blockade associated adverse events. CRA013442, Genome Sequence Archive, https://ngdc.cncb.ac.cn/gsa/browse/CRA013442. 10.1186/s13073-024-01285-9 PMC10799412 38243343

[73] Hu M. et al. Analysis scripts for “Gut Microbiome for Predicting Immune Checkpoint Blockade Associated Adverse Events”. GitHub; 2023. https://github.com/mnhu-work/IrAE_microbiome. 10.1186/s13073-024-01285-9 PMC10799412 38243343

