# Supplementary material for: Gut microbiome for predicting immune checkpoint blockade-associated adverse events
Source: Genome Med. 2024 Jan 19;16:16. doi: 10.1186/s13073-024-01285-9 (PMC10799412; doi:10.1186/s13073-024-01285-9)
Supplement: Supplementary file 1 — Additional file 1: Table S1. Sample information for downloaded raw data. Table S2. Clinical characteristics of reviewed studies. Table S3. Clinical characteristics of in-house cohorts. Table S4. Differential microbial species between irAEs and non-irAEs in the integrated anti-CTLA-4 datasets. Table S5. Differential microbial species between irAEs and non-irAEs in the integrated anti-PD-1/PD-L1 datasets. Table S6. Wilcox-rank sum test for species filtering between with blocking on the ‘Study’. Important species (all P value < 0.1) compared between irAEs and non-irAEs and between responders and non-responders, were showcased in the data sheet, respectively. Table S7. Fourteen Species and relative abundance for model classifier. Table S8. Wilcox-rank sum test for pathway from PICRUST2 output. Differential pathways (FDR < 0.005) were showcased in the data sheet. Table S9. Wilcox-rank sum test for EC number from PICRUST2 output. Differential EC (FDR < 0.005) were showcased in the data sheet. Table S10. Differential genes (FDR < 0.5) from the colon tissue RNA sequencing (N = 9) were showcased in the data sheet. Table S11. Differential KEGG pathways (FDR < 0.05) analyzed by gene set enrichment analysis (GSEA). [file 13073_2024_1285_MOESM1_ESM.zip › Additional file 1/Table.S2.docx]

Characteristics of reviewed studies

| Study | Tumor type | Treatment | 16S rRNA sequencing strategies | IrAE evaluation criterion | IrAE vs. nonIrAE definition |
| --- | --- | --- | --- | --- | --- |
| Cascone  (2020) | Lung cancer | Combined | 16S V4 rRNA gene sequencing on the Illumina MiSeq platform with 2 × 250-bp reads (Illumina, Inc.) | Common Terminology Criteria for Adverse Events (CTCAE) Version 5.0. | Enrolled patients were monitored for adverse events (AEs). AEs were treated as detailed in the protocol algorithm of toxicity management. We applied a Bayesian method to formally monitor the toxicity in the perioperative phase within each treatment arm. |
| Chau  (2021) | Lung cancer | anti-PD1 | 16S rRNA V3-V4 regions using the MiSeq Reagent Kit v3 (600 cycles) for 300bp pair-ends. | Common Terminology Criteria for Adverse Events (CTCAE) Version 5.0. | irAEs were defined as AEs consistent with an immune-mediated mechanism of action and requiring management with steroids or other immunosuppressants, and/or endocrine-targeted therapy for endocrinopathies |
| Hakozaki  (2020) | Lung cancer | anti-PD1/PDL1 | 16S rDNA V3–V4 hypervariable region using a paired-end, 2x250-bp cycle run on an Illumina MiSeq sequencing system and MiSeq Reagent Nano Kit version 2 | Common Terminology Criteria for Adverse Events (CTCAE) v4 | For irAE,the highestgrade toxicities during each therapy were recorded. Patients who experienced clinically relevant (≥grade 2) irAE compared with those with nonsevere irAE (grade 1 or absent). |
| Zhang  (2021) | Lung cancer | anti-PD1/PDL1 | 16S rDNA gene V3-V4 hypervariable region using a 2 × 300 pb paired-end run (MiSeq Reagent kit v3 (MS-102-3003)) on a MiSeq sequencer | \ | IrAEs were recorded with relative grades (0-4). For severity, no significant differences were observed in bacterial diversity between patients who experienced relevant clinical toxicities (≥grade 2) and patients with non-severe toxicities |
| McCulloch  (2022) | Melonoma cancer | anti-PD-1 | 16S rDNA V3-V4 on the NovaSeq System (Illumina) using the 2 × 150 base-pair (bp) paired-end protocol. | irAE severity was graded with CTCAE v5.0 | irAEs were defined as any clinical and/or laboratory event that occurred following initiation of anti-PD-1 therapy that was definitely linked to administration of anti-PD-1 therapy based on investigator assessment. Adverse events were considered irAEs based on mechanism of action and a prespecified list of terms developed by study investigators and grouped under the following broader terms: pneumonitis, colitis, hepatitis, nephritis, arthritis, thyroid (including hyperthyroidism, hypothyroidism and thyroiditis), adrenal (adrenal insufficiency), dermatologic (skin and subcutaneous disorders including rash, pemphigoid and vitiligo) and neurologic (Guillain-Barré syndrome, encephalitis and myasthenic syndrome). Infusion reactions without immunologic etiology were not included in this analysis. |
| Chaput  (2017) | Melonoma cancer | anti-CTLA4 | 16S rRNA V3–V4 using 454 pyrosequencing (Life Sciences, a Roche company, Branford, CT) and MiSeq (Illumina, Inc., San Diego, CA) technologies | \ | Recorded IrAEs using the identidication of the occurrence of ipilimumab-induced colitis, The diagnosis of ipilimumab-induced colitis was made in patients who had endoscopic signs of inflammation and no other cause of colitis, such as ischemia and infection (stool tests for bacterial pathogens and Clostridium difficile toxin had to be negative. |
| Dubin  (2015) | Melonoma cancer | anti-CTLA4 | 16S rRNA gene V4–V5 region on an Illumina MiSeq platform using 2X250 nucleotide paired-end sequencing | CTCAE, version 4.0, and grading on the terms diarrhoea and colitis | . Patients were assigned a colitis score based on the following: no diarrhoea (score 0), grade 1 diarrhoea (score 1), grade 2 diarrhoea (score 2), grade 2 diarrhoea and/or grade 2 colitis (all 3 cases had both grade 2 diarrhoea and grade 2 colitis) (score 3), grade 3 diarrhoea and/or grade 3 colitis (1 case with both grade 3 diarrhoea and grade 3 colitis) (score 4). |
| Baruch  (2022) | Melonoma  cancer | FMT | The extracted DNA underwent a Polymerase Chain Reaction (PCR) using 515-Forward  and 806-Reverse primers for the V4 variable region of the bacterial 16S rRNA gene. | - | - |

Note: Each study is indicated by first author and year of publication. For each study, cancer type, type of ICI therapy, IrAE criteria utilized, definition for IrAE and nonIrAE, sequencing technology used are shown.
