# Supplementary material for: Gut microbiome for predicting immune checkpoint blockade-associated adverse events
Source: Genome Med. 2024 Jan 19;16:16. doi: 10.1186/s13073-024-01285-9 (PMC10799412; doi:10.1186/s13073-024-01285-9)
Supplement: Supplementary file 1 — Additional file 1: Table S1. Sample information for downloaded raw data. Table S2. Clinical characteristics of reviewed studies. Table S3. Clinical characteristics of in-house cohorts. Table S4. Differential microbial species between irAEs and non-irAEs in the integrated anti-CTLA-4 datasets. Table S5. Differential microbial species between irAEs and non-irAEs in the integrated anti-PD-1/PD-L1 datasets. Table S6. Wilcox-rank sum test for species filtering between with blocking on the ‘Study’. Important species (all P value < 0.1) compared between irAEs and non-irAEs and between responders and non-responders, were showcased in the data sheet, respectively. Table S7. Fourteen Species and relative abundance for model classifier. Table S8. Wilcox-rank sum test for pathway from PICRUST2 output. Differential pathways (FDR < 0.005) were showcased in the data sheet. Table S9. Wilcox-rank sum test for EC number from PICRUST2 output. Differential EC (FDR < 0.005) were showcased in the data sheet. Table S10. Differential genes (FDR < 0.5) from the colon tissue RNA sequencing (N = 9) were showcased in the data sheet. Table S11. Differential KEGG pathways (FDR < 0.05) analyzed by gene set enrichment analysis (GSEA). [file 13073_2024_1285_MOESM1_ESM.zip › Additional file 1/Table.S3.docx]

| Characteristic | SH Cohort  (Amplicon Dataset) | JS Cohort  (Metagenome Shotgun Dataset) |
| --- | --- | --- |
| **Tumor Type** |  |  |
| ColorectalCa | 19 | 4 |
| GastricCa | 32 | 5 |
| LungCa | 14 | 30 |
| Others | 0 | 11 |
| **Sex** |  |  |
| Female | 17(26.2%) | 11(22.0%) |
| Male | 48(73.8%) | 39(78.0%） |
| **Age, years**, median ± SD | 66±12.07 | 65.5±13.17 |
| **BMI,**  median ± SD | 21.50±3.21 | 26.02±5.14 |
| **ICB type** |  |  |
| Anti-PD1/PD-L1 | 65 | 50 |
| **IrAE Type** |  |  |
| Digestive system | 2 | 5 |
| Hemopoietic and blood system | 9 | 7 |
| Skin | 2 | 2 |
| Urinary system | 3 | 0 |
| Others | 7 | 2 |
| **IrAE Severity** |  |  |
| None/Mild | 52 | 41 |
| Moderate/Severe | 13 | 9 |

**Clinical characteristics of in-house cohorts.**
