# Supplementary material for: Gut microbiome for predicting immune checkpoint blockade-associated adverse events
Source: Genome Med. 2024 Jan 19;16:16. doi: 10.1186/s13073-024-01285-9 (PMC10799412; doi:10.1186/s13073-024-01285-9)
Supplement: Supplementary file 2 — Additional file 2: Fig S1. Alpha and beta diversity analysis in each cohort. Fig S2. Confounder analysis for model construction. Fig S3. Wilcoxon rank-sum test for the relative abundance comparison within 14 model species. Fig S4. Model evaluation in machine learning. Fig S5. Integrated analysis with colon tissue RNA sequencing and 16S rRNA amplicon sequencing. Fig S6. Qualitative analysis on menaquinone in key microbes. [file 13073_2024_1285_MOESM2_ESM.zip › Additional file 2/Supplementary Figure legends.docx]

**Additional file 2**

**Fig.S1**

(A). Alpha diversity evaluation between irAEs and non-irAEs using Shannon Index in each single study, a two-sided Wilcoxon Rank-Sum test was used for comparison. The group (irAEs and non-irAEs) is indicated by different colors.

(B). Statistical analysis was conducted between the irAE status and the tumor type, Chi-square test.

(C). β diversity (principal coordinate analysis with Bray-Curtis dissimilarity) based on the species abundances in the irAEs and non-irAEs groups for single study. The group (irAEs and non-irAEs) is indicated by different colors.

(D). Alpha diversity evaluation among studies, a two-sided Wilcoxon Rank-Sum test was used for comparison. The group (study) is indicated by different colors.

(E). Original Principal Coordinates Analysis (PCoA) analysis colored by irAEs. The PCoA was conducted with Bray-Curtis dissimilarity based on the species abundances in the individuals. The group (irAEs and non-irAEs) is indicated by different colors.

(F). Adjusted PCoA (after adjusting covariates with tumor type) analysis colored by irAEs.

The PCoA was conducted with Bray-Curtis dissimilarity based on the species abundances in the individuals. The group (irAEs and non-irAEs) is indicated by different colors.

**Fig.S2.** Confounder analysis for model construction.

1. Potential confounding of individual microbial species associations by patient demographics and technical factors. Variance explained by irAEs status (irAEs versus non-irAEs) is plotted against variance explained by different putative confounding factors for individual microbial species. Each species is represented by a dot proportional in size to its abundance; differential microbial markers identified in the meta-analysis are highlighted in red. For the confounder analysis, factors with continuous values were discretized into quartiles and the BMI was split into lean/overweight/obese according to conventional cutoffs. The variance explained by irAEs status was computed for all data. The variance explained by different confounding factors was computed using all samples for which data were available.
2. Principal coordinate analysis with Bray-Curtis dissimilarity based on the species abundances in the Female and Male. The group (Female and Male) is indicated by different colors.
3. Principal coordinate analysis with Bray-Curtis dissimilarity based on the species abundances in the different age level (Using 60 as the cutoff of age). The group (>=60 and <60) is indicated by different colors.

**Fig.S3. (A-N).** The relative abundance of representative 14 model species comparison in irAEs and non-irAEs using integrated datasets. Each dot represented one sample. P values were computed using a two-sided Wilcoxon rank-sum test. (O). A list of the name of 14 model species.

**Fig.S4.** (A-D). Precision-recall curve (PRC) assessed for statistical modeling in the training set as well as validation cohorts.

(E). Validation performance of reconstructed model using Chau et al.

(F). The AUC of the optimized models constructed with the P<0.05 features for distinguishing non-irAEs from irAEs. Mean AUC and standard deviation of stratified 10-fold cross-validation were shown.

**Fig.S5.** Potential mechanism deduction from an external cohort integrating colon tissue RNA sequencing and 16S rDNA amplicon sequencing.

(A). Differential genes (FDR<0.5) between RF-score high and RF-score low; red color indicates upregulation and blue color indicates downregulation in RF-score low group.

(B). Gene set enrichment analysis (GSEA) for KEGG pathways enriched (FDR < 0.05) in RF-score low group(right) or RF-score high group(left).

**Fig.S6.** (A). The top 10 species identified with correlation test between microbial abundance and menaquinone pathways using the JS WGS dataset.

(B-E) Analysis of the menaquinone-6 (MK-6) using high-performance liquid chromatography among (B)Supernatant from *Parabacteroides merdae(PM)*, (C) Brain Heart Infusion (BHI) Culture Medium (*PM* culture medium), (D) Supernatant from *Lactobacillus salivarius (LS)*, and (E) deMan Rogosa Sharpe (MRS) Culture Medium (*LS* culture medium)
