# Supplementary figures and images for: Gut microbiome for predicting immune checkpoint blockade-associated adverse events
Source: Genome Med. 2024 Jan 19;16:16. doi: 10.1186/s13073-024-01285-9 (PMC10799412; doi:10.1186/s13073-024-01285-9)

Fig.S1

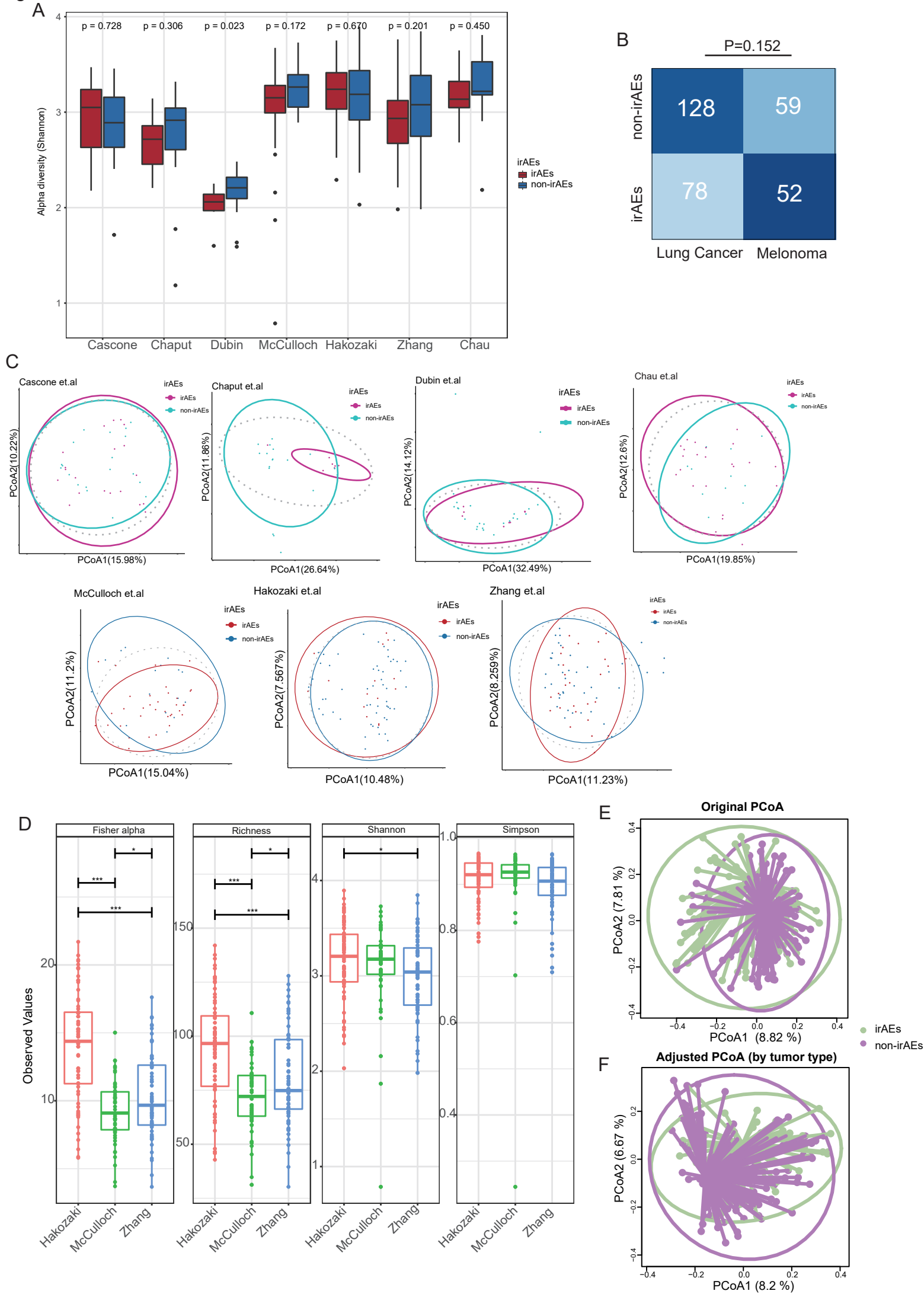

Fig.S2

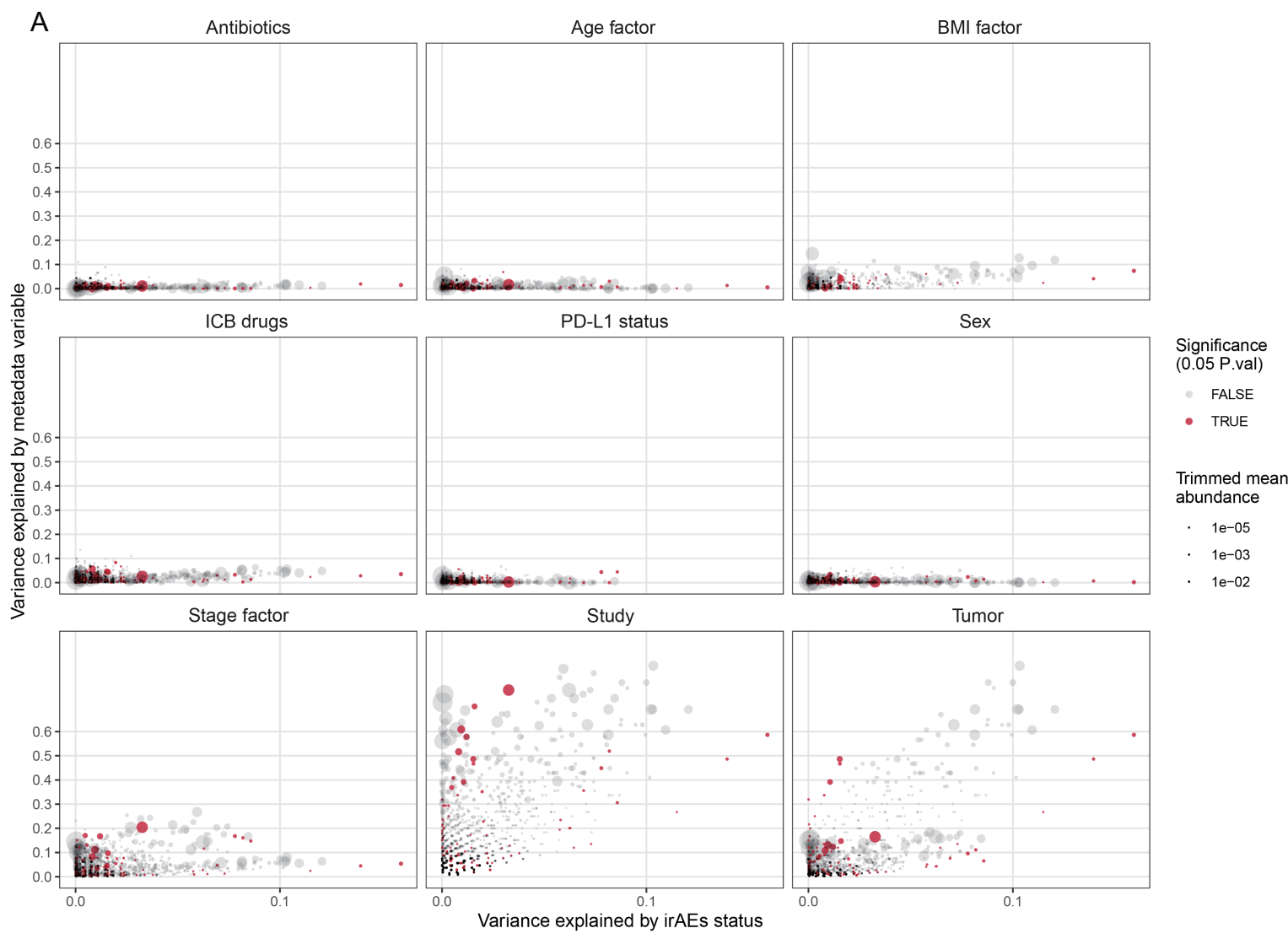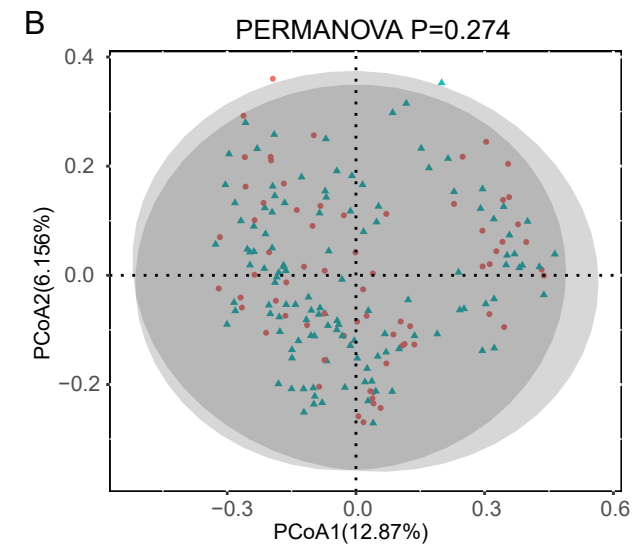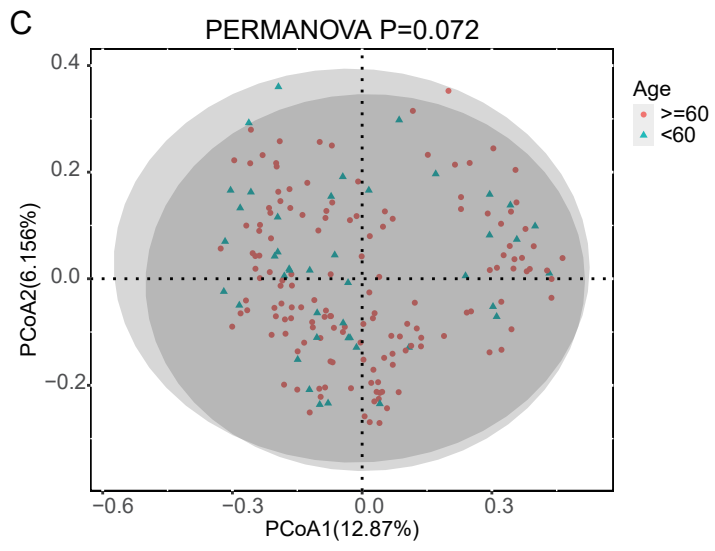

Fig.S3

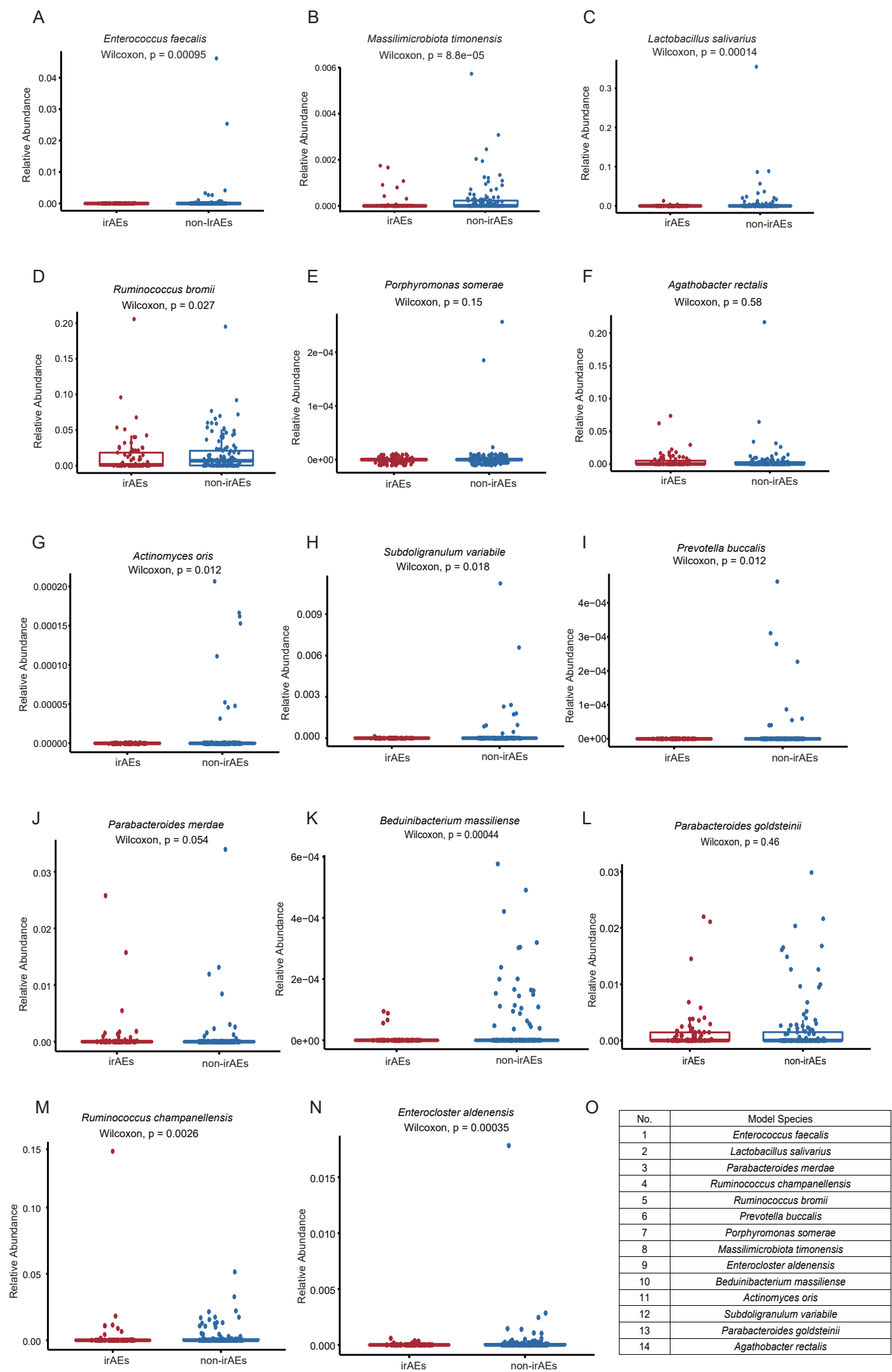

Fig.S4

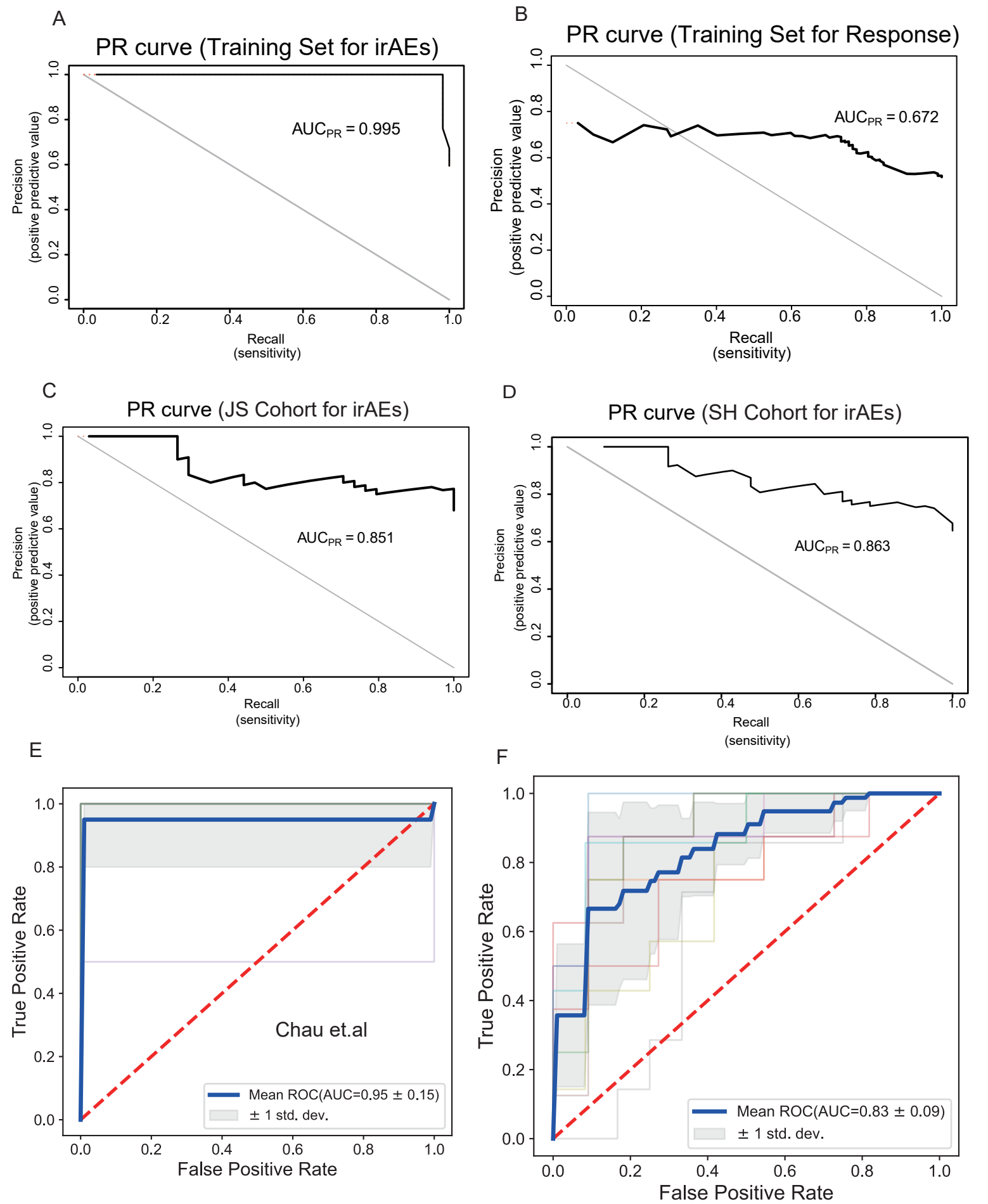

Fig.S5

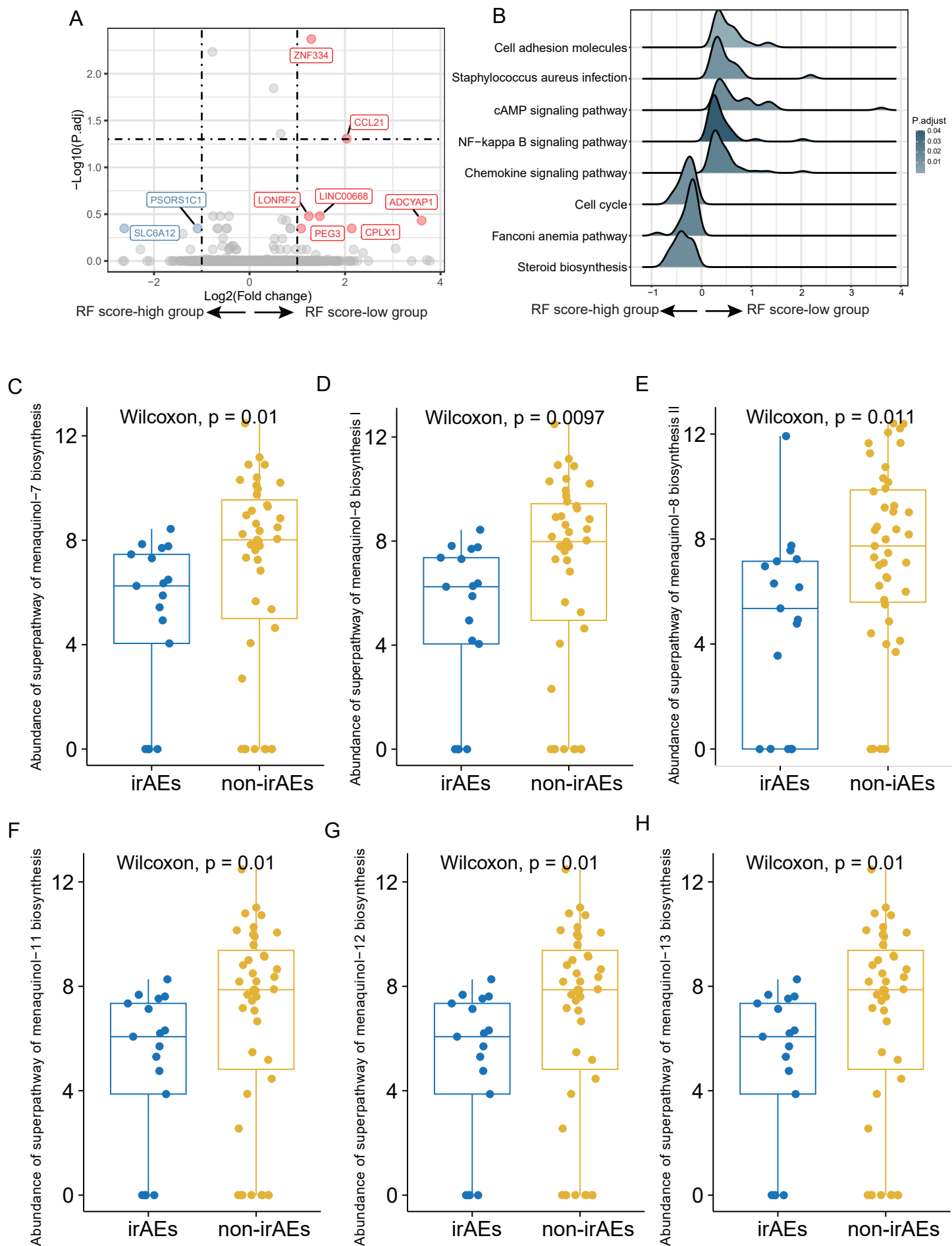

Fig.S6

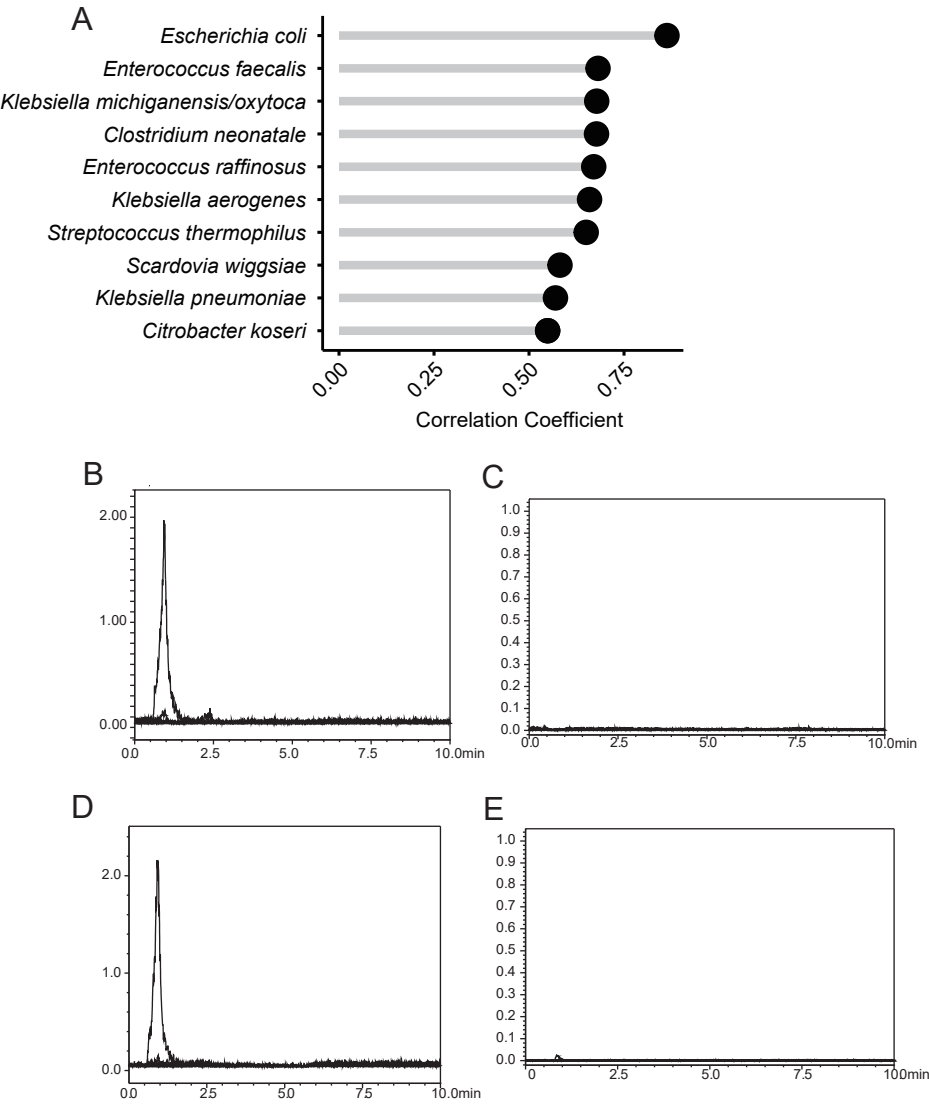

Supplement: Supplementary file 2 — Additional file 2: Fig S1. Alpha and beta diversity analysis in each cohort. Fig S2. Confounder analysis for model construction. Fig S3. Wilcoxon rank-sum test for the relative abundance comparison within 14 model species. Fig S4. Model evaluation in machine learning. Fig S5. Integrated analysis with colon tissue RNA sequencing and 16S rRNA amplicon sequencing. Fig S6. Qualitative analysis on menaquinone in key microbes. [file 13073_2024_1285_MOESM2_ESM.zip › Additional file 2/Supplement_Figure.pdf]
